# Nitrogen and Phosphorus Limitation over Long-Term Ecosystem Development in Terrestrial Ecosystems

**DOI:** 10.1371/journal.pone.0042045

**Published:** 2012-08-03

**Authors:** Duncan N. L. Menge, Lars O. Hedin, Stephen W. Pacala

**Affiliations:** 1 Department of Ecology and Evolutionary Biology, Princeton University, Princeton, New Jersey, United States of America; 2 National Center for Ecological Analysis and Synthesis, Santa Barbara, California, United States of America; 3 Department of Biology, Stanford University, Stanford, California, United States of America; Utrecht University, The Netherlands

## Abstract

Nutrient limitation to net primary production (NPP) displays a diversity of patterns as ecosystems develop over a range of timescales. For example, some ecosystems transition from N limitation on young soils to P limitation on geologically old soils, whereas others appear to remain N limited. Under what conditions should N limitation and P limitation prevail? When do transitions between N and P limitation occur? We analyzed transient dynamics of multiple timescales in an ecosystem model to investigate these questions. Post-disturbance dynamics in our model are controlled by a cascade of rates, from plant uptake (very fast) to litter turnover (fast) to plant mortality (intermediate) to plant-unavailable nutrient loss (slow) to weathering (very slow). Young ecosystems are N limited when symbiotic N fixation (SNF) is constrained and P weathering inputs are high relative to atmospheric N deposition and plant N:P demand, but P limited under opposite conditions. In the absence of SNF, N limitation is likely to worsen through succession (decades to centuries) because P is mineralized faster than N. Over long timescales (centuries and longer) this preferential P mineralization increases the N:P ratio of soil organic matter, leading to greater losses of plant-unavailable N versus P relative to plant N:P demand. These loss dynamics favor N limitation on older soils despite the rising organic matter N:P ratio. However, weathering depletion favors P limitation on older soils when continual P inputs (e.g., dust deposition) are low, so nutrient limitation at the terminal equilibrium depends on the balance of these input and loss effects. If NPP switches from N to P limitation over long time periods, the transition time depends most strongly on the P weathering rate. At all timescales SNF has the capacity to overcome N limitation, so nutrient limitation depends critically on limits to SNF.

## Introduction

Over the past few decades an elegant conceptual model of long-term ecosystem development has emerged that focuses on the roles of nitrogen (N) and phosphorus (P) in shaping terrestrial ecosystem dynamics. This conceptual model concerns the development of ecosystems during primary succession–i.e., after a catastrophic disturbance such as a volcanic eruption or glacial retreat–over time periods extending to millions of years in the absence of another catastrophic disturbance. A basic component of the model is the contrast between abiotic N versus P inputs as soils develop. P inputs decline over time because P weathers out of rocks, whereas abiotic N inputs do not change much with soil age because they come primarily from the atmosphere (although some rocks contain substantial amounts of N [1]). Declining P inputs have been proposed to produce a “terminal steady state” of P deficiency [2], [3].

These abiotic P versus N input dynamics have been associated with a transition in nutrient limitation to net primary production (NPP), with NPP being N limited on young soils and P limited on old soils. For example, fertilization studies in Hawaiian montane rainforests show that forest growth is N limited on young (300 year old) soils, co-limited by N and P on intermediate (20,000 year old) soils, and P limited on old (4.1 million year old) soils [4]. While direct fertilization tests are lacking, data on nutrient distributions in plants and soils from long-term chronosequences in New Zealand [5], Arizona [6], [7], and elsewhere [8] have been interpreted to be consistent with a transition to P limitation as soils age.

However, a number of issues complicate the input side of the nutrient limitation picture. The first concerns biotic N inputs via biological N fixation, which is at least as important as abiotic N deposition in most ecosystems. At the young end of the spectrum, many researchers [3], [9]–[12] have pointed out that the scarcity of N in young soils often leads to the dominance of woody symbiotic N fixers (hereafter, “N fixers”). N fixers can often fix more than enough N to meet their own demand [13] (but see [14]) and their litter adds substantial N to the soil, so N limitation to NPP might actually be uncommon when N fixers are sufficiently common. Indeed, P has been shown to limit symbiotic N fixation (hereafter, SNF) and presumably NPP in some young Alaskan forests [15]. Thus, when N fixers are abundant and actively fixing N, NPP might be more likely to be P limited or co-limited than N limited.

At the old end of the age spectrum, P inputs via atmospheric deposition of dust and aerosols become at least as important as P inputs from rock weathering. Atmospheric P deposition likely depends on climate patterns and the properties of upwind ecosystems [16]–[18] rather than soil age. Furthermore, tectonic uplift [19] or erosion could replenish parent material P such that weathering P inputs remain significant even at the terminal steady state. If there is a continual P input in addition to a continual N input, is a transition to P limitation inevitable? Apart from some very old soils [3], [5], [6], unpolluted old-growth forests in temperate and boreal regions are typically thought to be N limited [20]–[22]. Are the soils on which these forests grow simply not old enough to have reached the terminal P-limited steady state? Or is their steady state N limited?

So far this discussion has focused on N and P inputs, but losses can play an equally vital role in determining nutrient limitation. In particular, losses of plant-unavailable nutrients are essential to the maintenance of nutrient limitation [23]–[27]. One such loss vector–leaching of dissolved organic forms of N or P (DON or DOP)–has been implicated in maintaining N limitation in old temperate forests [22], [23], [28] and P limitation in old tropical forests [24]. Other losses of plant-unavailable nutrients such as physical erosion or hydrologic losses of particulate N or P might also be important, but for brevity we often refer to losses of plant-unavailable nutrient as DON or DOP losses.

These examples illustrate that different states–N limitation versus P limitation–might be possible at different times during ecosystem development. Young forests might be N limited because rocks have little N, or P limited because N fixers dominate the communities and fix as much N as they need. Old forests might be P limited because the majority of P has weathered out of rocks and DOP losses are high enough, N limited because they are not old enough to reach the P-limited steady state, or N limited because sufficiently large dust P inputs or DON losses produce an N-limited terminal steady state.

These lines of evidence imply a diverse natural history of ecosystem development with several possible trajectories, and thus identify the need for a broader model with the quantitative rigor to develop alternative testable hypotheses. Under what conditions should N limitation and P limitation prevail? If there are transitions between these different ecosystem states, when do they occur? Which ecosystem properties and processes influence why and when these transitions occur, and which are less relevant? What effect do symbiotic N fixers have on these states and transitions between them? These questions about ecosystem development concern fundamental ecosystem properties that, in addition to being interesting in their own right, determine the extent to which ecosystems themselves will help mitigate anthropogenic environmental impacts. As atmospheric reactive N levels are increasing [29], [30], the ability of ecosystems to sequester additional N and carbon (C) depends on whether N, P, or both limit production. N-limited ecosystems would respond to N deposition by absorbing additional N and CO_2_ [31] and mitigating global warming, whereas P-limited ecosystems might not soak up additional N or CO_2_ [32], and would likely exhibit negative effects of N saturation such as decreases in stream and lake water quality, downstream eutrophication, and increased emissions of NO and the greenhouse gas N_2_O [33]–[36].

In the present work we develop and analyze a dynamical model to investigate the questions posed in the previous paragraph. In particular, we take advantage of the natural timescale separation inherent to biogeochemical processes [26], [37] to solve for the transient and equilibrium dynamics of plant biomass, litter N and P, soil organic matter N and P, and plant-available N and P. Additionally, we derive the conditions under which N limitation and P limitation to NPP are expected and the times at which transitions between N and P limitation occur. We focus on three periods in ecosystem development: the initial stages of primary succession, the late stages of succession on relatively young soils, and the terminal steady state on geologically old soils. Our purposes in this work are three-fold. First, given a set of known biogeochemical mechanisms, we investigate possible nutrient limitation trajectories, looking specifically for counterintuitive dynamics that have received little attention in the literature. Second, we provide a quantitative framework in which to examine where these different nutrient limitation trajectories might occur. Third, we present an analytical multiple timescale framework that could render multitudes of other ecosystem-level questions mathematically tractable.

## Methods

### Model Description

**Figure 1:**
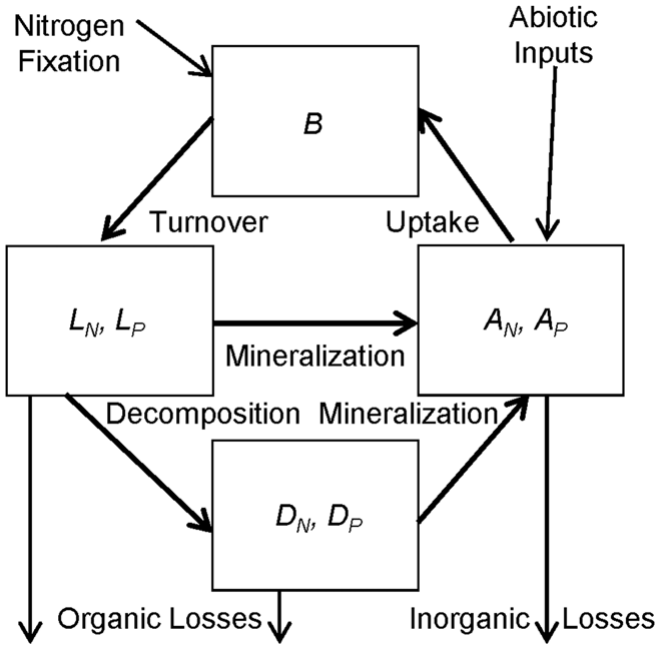
Model ecosystem described in **equations 1**–**7**. Living plant biomass (*B*) contains carbon, nitrogen (N), and phosphorus (P) in a fixed stoichiometric ratio. When plant biomass turns over it becomes litter. The model keeps track of litter N (*L_N_*) and P (*L_P_*) separately. Some litter is decomposed into plant-unavailable soil organic matter (SOM; *D_N_*, *D_P_*), some is mineralized to plant-available soil nutrients (*A_N_*, *A_P_*), and some is lost from the system. Some SOM is mineralized into plant-available nutrients, and some is lost. Plant-available nutrients come from mineralization and external inputs, are taken up by plants, and are lost from the ecosystem. Symbiotic N fixation enters directly into the plant pool. We use “mineralization” as shorthand for “mineralization and depolymerization” to indicate the conversion of nutrient to plant-available form, which can include some organic forms.

The basic model we use is an extension of previous work [25], [26], [38]–[40], which draws on earlier models [27], [41]–[43]. The present version tracks changes through time in plant biomass carbon (*B*); organically bound, plant-unavailable N and P in litter (*L_N_*, *L_P_*); organically bound, plant-unavailable N and P in soil organic matter (SOM, which is distinct from litter in that it is sufficiently decomposed to the point where its original source is uncertain [44]; *D_N_*, *D_P_*); and plant-available N and P in the soil (*A_N_*, *A_P_*). Units of each variable are element (C, N, or P) mass per area [kg ha^−1^]. We track N and P independently in litter and SOM because marked variation in soil N:P occurs throughout ecosystem development [5], [45] (also see Discussion). In addition to inorganic N (e.g., nitrate and ammonium), plants can access N in some organic molecules such as amino acids [46], [47]. We know of no evidence that plants directly take up organic P, but small P-containing molecules like nucleotides are likely candidates, and in effect plants can access some organic P via root phosphatases [47]. Therefore, we distinguish *A_N_* and *A_P_* from the other soil N and P pools by whether or not plants can acquire them rather than by their chemical form. The model, cartooned in Fig. 1, is defined by the equations:


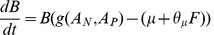
(1)


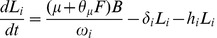
(2)


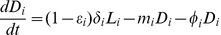
(3)


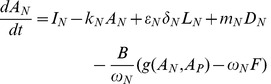
(4)


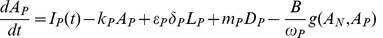
(5)


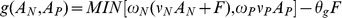
(6)


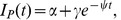
(7)

where the subscript *i* indicates N or P. Due to the nature of our questions we assume in this model that NPP is limited by N, P, or both, although other factors such as water, light, and top-down control also limit NPP in many real ecosystems. This assumption appears in the plant growth function *g*(*A_N_*, *A_P_*), which is a Liebig’s law function specifying that NPP increases with plant-available N, P, or at the exact ratio of plant N:P demand, both [41], [48]. Although environmental factors such as moisture are known to influence most of the processes in our model [49], [50], we do not include them explicitly here for the sake of simplicity. They appear implicitly in this model in that different sites would be represented by different parameter values, such as faster weathering [51] in wetter ecosystems compared to drier ecosystems.

Growth via N or P limitation depends on nutrient acquisition via uptake (*ν_i_*) and SNF (*F*) and the creation of new biomass C as mediated by nutrient use efficiencies (*ω_i_*). Plant mortality and tissue turnover (*μ + θ_μ_ F*) transfer N and P from plants to litter. SNF carries costs associated with both growth (*θ_g_*) and turnover (*θ_μ_*) relative to uptake of soil N [21], [39], [40], [52], [53]. Litter N and P decompose at rates *δ_i_*, with fractions *ε_i_* for each being mineralized and the remaining fractions (1– *ε_i_*) becoming SOM. SOM N and P are mineralized at rates *m_i_*. There are losses from all soil pools–*h_i_* from litter, *φ_i_* from SOM, and *k_i_* from plant-available pools–which can include leaching of dissolved and particulate nutrients (for all), erosion (for all), and gaseous losses (for plant-available N, e.g., from denitrification or nitrification). In this work we do not distinguish between different types of loss vectors from a given pool–such as leaching versus erosion of SOM or leaching versus gas loss of plant-available N–but include them all together. Abiotic inputs of N and P (*I_N_* and *I_P_*(*t*)) enter the plant-available soil pools. The definitions, units, and base values of all parameters are given in Table 1. Throughout this work we are careful to distinguish the term “rate”–a constant with units time^−1^ such as *δ_i_*, *φ_i_*, or *ψ*–from “flux,” which has units of mass area^−1^ time^−1^ and may be a constant such as *I_N_* or a variable such as *I_P_*(*t*).

**Table 1:** Variables, functions, and parameters.

Symbol	Definition	Units	Restrictions	Value or N value	P
*B*	Plant biomass C	kg C ha^−1^	≥0		
*L_i_*	Litter soil nutrients	kg *i* ha^−1^	≥0		
*D_i_*	SOM nutrients	kg *i* ha^−1^	≥0		
*A_i_*	Plant-available soil nutrients	kg *i* ha^−1^	≥0		
*g*(*A_N_*,*A_P_*)	Plant growth function	yr^−1^	≥0		
*ω_i_*	Nutrient use efficiencies	kg C kg *i* ^−1^	>0	50	600
*ν_i_*	Nutrient uptake parameters	ha kg C^−1^ yr^−1^	>0	2	20
*F*	N fixation parameter	kg N kg C^−1^ yr^−1^	≥0	0	…
*µ*	Biomass turnover rate	yr^−1^	>0	0.5	…
*θ_g_*	Growth cost of N fixation	kg C kg N^−1^	0−(*ω_N_−θ_μ_*)	5	…
*θ_μ_*	Turnover cost of N fixation	kg C kg N^−1^	0−(*ω_N_−θ_g_*)	5	…
*δ_i_*	Litter decomposition rates	yr^−1^	>0	4	4
*h_i_*	Litter loss rates	yr^−1^	>0	0.001	0.001
*ε_i_*	Proportions of litter decomposition mineralized	unitless	0–1	0.4	0.5
*m_i_*	SOM decomposition rates	yr^−1^	>0	0.03	0.04
*φ_i_*	SOM loss rates	yr^−1^	>0	0.001	0.001
*I_i_*	Plant-available nutrient input fluxes	kg *i* ha^−1^ yr^−1^	>0	2	*α + γ*e*^−ψt^*
*Α*	P input flux from dust	kg P ha^−1^ yr^−1^	>0	…	0.01
*γ*	P weathering flux from virgin rock	kg P ha^−1^ yr^−1^	>0	…	0.4
*ψ*	P weathering rate	yr^−1^	>0	…	0.00025
*k_i_*	Plant-available nutrient loss rates	yr^−1^	>0	6	4

Table notes: *i* refers to nitrogen (N) and phosphorus (P). C: carbon. SOM: soil organic matter. Parameters are from [40], except for *δ_i_* and *m_i_* (adapted from *m* in [40] to account for two soil organic pools per nutrient here rather than one), *h_i_* (chosen to equal *φ_i_*), *ε_i_* (reasonable guesses), *k_i_* (adjusted to reflect greater N than P loss due to greater soil mobility), and P input parameters (chosen to equal *I_P_* in the previous work on young soils but for weathering to proceed at a relatively rapid rate).

The structure of equations 1–7 differs from those in [40] in two key ways. First, plant-unavailable N and P in the soil are now divided into two pools each–litter and SOM–whereas previous models included a single plant-unavailable pool per nutrient. There is ample evidence that organic matter is heterogeneous and decomposes at multiple timescales [54], [55]. Second, to capture P input dynamics at long timescales, *I_P_*(*t*) includes both a constant flux (e.g., dust deposition, uplift, and erosion [19], *α*) and a flux that decreases with time (e.g., weathering, *γe^−ψt^*, with the weathering rate *ψ*), as in [56]. True weathering dynamics might involve multiple timescales corresponding to P-containing minerals with different weathering rates, but we included only a single rate for simplicity. See Appendix S1 for a derivation of the P input term. Inclusion of inputs from weathering of rock N, which is important in some areas [1], would be a simple matter of altering *I_N_* to be analogous to *I_P_*(*t*).

Because our primary focus in this paper is on timescales longer than a few months, we do not consider a number of short-term processes. For example, immobilization of N onto litter occurs in fresh litter with a high C:N ratio, but because there must be net mineralization over the timescales we consider [57], we ignore immobilization, in effect averaging over short timescales. As another example, rapid fluctuations in soil moisture are known to affect soil N [49], [50] and P [58] dynamics, but our approach here is to treat our parameters as averages over these shorter timescales. To test whether this averaging of rapid fluctuations might unduly influence our longer timescale results, we conducted simulations where some of our parameter values (those likely to be influenced by soil moisture) fluctuated rapidly. For the variety of conditions we investigated, the effects on longer timescales were minimal (see Fig. S2).

### Model Analysis

Although the model structure in the present paper differs from [40] in only two ways, our questions and analyses are entirely different. Menge et al. [40] examined how different degrees of regulation of SNF affected ecosystem properties. In the present paper we are concerned with how myriad ecosystem processes (including SNF, but also including many others) affect the development of N versus P limitation over long timescales. The analyses we use in the present paper build on [26], which examined how different loss types affect N limitation over multiple timescales. However, because [26] did not include P, it did not examine limitation by N versus P, which is the main focus of the present paper.

Our full model (equations 1–7) is sufficiently complicated that many of our analytical goals, such as solving for the transition times between N and P limitation, appear intractable. However, the natural diversity of timescales within biogeochemical systems allows solutions for the transient dynamics via the techniques of timescale separation [26], [59]–[61]. The basic idea of timescale separation is that when some ecosystem components (e.g., litter) change much more rapidly than others (e.g., weatherable P in rock), one can solve for the fast dynamics (e.g., litter turnover) assuming that the slow components (e.g., the P weathering input) are effectively constant, and solve for the slow dynamics assuming that the fast components are at their quasi equilibria. We distinguish quasi equilibria, which are the equilibria of the fast components on their timescales, from equilibria (of the slowest timescale, i.e., the terminal steady state) because the quasi equilibria depend on slower variables that are dynamic at the longer timescales. With timescale separation, despite the overall complexity of equations 1–7, we can solve approximately for how plant biomass, plant-available soil nutrients, litter, and SOM N and P change over time, which nutrient is limiting at any given time, and when limitation switches from N to P.

The first step in timescale separation analysis is to determine the different timescales based on empirical knowledge of the processes. We analyzed equations 1–7 with three distinct timescales (short, intermediate, and long), although our results indicate that more than three exist (i.e., there are two distinct timescales within the “short timescale” of our analyses). Our short timescale tracks changes in plant-available and litter N and P, assuming that plant biomass, SOM N and P, and weathering P inputs are constant. Nitrate, ammonium, and plant-available organic N (e.g., amino acids) turn over within hours to days in grasslands and forests [46], [62]–[66]. Litter N and P turn over on the order of months to a few years [55], [67]. Our intermediate timescale tracks changes in plant biomass over years to decades (averaging foliar, root, and wood turnover rates for different plant types), assuming that plant-available and litter N and P remain at their quasi equilibria and that SOM N and P and weathering P inputs are constant. Assuming that plant-available and litter N and P remain at their quasi equilibria does not mean they remain constant in time because the quasi equilibria depend on plant biomass, which changes over the intermediate timescale. Our long timescale tracks changes in SOM N and P and P inputs over centuries to millennia and beyond, assuming that plant-available and litter N and P and plant biomass are all at their quasi-equilibria.

After breaking the system into three timescales, we analyzed the short timescale assuming that plant biomass, SOM N and P, and weathering P inputs are constant. This tells us how litter N and P and plant-available N and P change in the first hours to years following a disturbance. The transient dynamics depend on whether N or P limits plant growth, so we solved both the N-limited and P-limited cases. To determine whether N or P limits plant growth at the beginning of the short timescale, we simply evaluated the growth equation (equation 6) with the starting conditions (i.e., the post-disturbance amounts of litter and plant-available N and P). If the limiting nutrient was different at the beginning versus the end of the short timescale, we solved for the transition time *t_N→P_* or *t_P→N_*, which is defined as the time at which N and P co-limit growth. For the case of a transition from N limitation to P limitation, we use the expressions from when plants are N limited (*g*(*A_N,N_*(*t_N→P_*)) * =  g*(*A_P,N_*(*t_N→P_*))), and vice versa (*g*(*A_P,P_*(*t_P→N_*)) * =  g*(*A_N,P_*(*t_P→N_*))) for a transition from P limitation to N limitation. The first subscript in *A_P,N_* indicates the variable (in this case plant-available P) and the second subscript indicates the nutrient limitation case (in this case N limitation).

Switching from the short to the intermediate timescale (or the intermediate to the long) introduces a small discontinuity, or break, in the transient dynamics. Litter N and P and plant-available N and P approach their quasi equilibria asymptotically, meaning they never actually reach it, whereas at the intermediate timescale we assume they are exactly at their quasi equilibria. Deciding when to transition to the intermediate timescale involves balancing the jump to the quasi equilibrium–a better approximation the longer we wait–against the assumption that plant biomass remains constant–a worse approximation the longer we wait. We chose to transition to the intermediate timescale when litter and the limiting nutrient both came within 1% of their quasi equilibria. The intermediate timescale dynamics are then approximately linear (see Results section), so they can be solved assuming that litter and plant-available N and P are at their quasi equilibrium at that SOM N and P and weathering P inputs are constant. As above, we solve for N-limited and P-limited plant biomass, determine limitation at the beginning and end of the timescale, and solve for the time at which limitation switches.

Transitioning to the long timescale also involves a discontinuity, and we made the same assumption that the transition occurs when plant biomass comes within 1% of its quasi equilibrium. The long timescale dynamics and switches in limitation can then be determined as above. The transient solutions for each timescale will demonstrate the global stability at that timescale–e.g., stable if they approach an asymptote and unstable if they do not.

Timescale separation yields approximate solutions to the full system, and thus the results are only as good as the approximation. To check timescale approximations we numerically integrated the full system using the ode45 function in MATLAB 7.60. R2008a. For timescale approximations and numerical integrations we did not allow negative variable values because they are biologically meaningless. If timescale separation yields a decent approximation of the true model dynamics given by the numerical simulations, our analysis lends insight into the controls on transient dynamics at each timescale.

“Time” in the timescale approximations is the time since the last event that altered the variables in any way other than the system description. For example, a landslide that results in a rapid loss of soil and plants from a given site would reset all the variables; “time” would begin again at zero and the full set of timescales would be evaluated. Setting the initial condition for the time-dependent P input (*γ*, which depends on the initial amount of weatherable material; see Appendix S1) would be the trickiest initial conditions to parameterize in this case. A nitrogen fertilization event (say, with calcium nitrate) would only reset the plant-available N pool, but again, time would restart at zero and the full set of timescales would be evaluated.

### Parameter Values and Assumptions

Because our primary results are analytical, they do not depend on the exact parameter values beyond the general bounds of timescale separation. That is, our analysis would yield strange results if rock weathering were faster than litter decomposition, but this is an unrealistic situation [51], [67] that is beyond the scope of real ecosystems. For simulations we chose to use a reasonable parameter set for forests (Table 1). We used the same set of parameters as in [40], which explains the sources of each value, with exceptions noted in the Table 1 notes. Our explicit results are often too bulky to be informative, but simplifications based on assumptions about relationships between the parameters are quite informative. For instance, we assume that litter decomposition rates are much higher than litter loss rates (*δ_i_ >> h_i_*). These simplifying assumptions are listed in Table 2 along with reasons for the assumptions and likely scenarios in which they do not hold.

**Table 2:** Parameter assumptions for analytical simplifications.

Assumption	Reasons/references	Exceptions
*δ_i_*, *δ_i_*(1*– ε_i_*) *>> h_i_*	Recycling exceeds losses^1^	Heavy soil disturbance
*Bν_i_ >>k_i_*	Recycling exceeds losses^1^	Low *B* (after disturbance)
*m_i_ >> φ_i_*	Recycling exceeds losses^1^	Heavy soil disturbance
*φ_i_ >> ψ*	Weathering is very slow^2^	Rapid weathering rate
*ω_P_>> ω_N_*	Biological stoichiometry^3^	
*ε_P_> ε_N_*	Litter biochemistry^4^	Common ground fires
*m_P_> m_N_*	SOM biochemistry^4^	Common ground fires
*δ_P_ ≈ δ_N_*	Parameter definition^5^	
*h_P_ ≈ h_N_*	Same material^6^	Common ground fires
*φ_P_ ≈ φ_N_*	Same material^6^	Common intense fires
*m_i_D_i_ >>* |*I_i_ – k_i_A_i,i_*|	Recycling exceedsinputs/losses^1^	Heavy pollution, Low *D*

Table notes and references: The subscript *i* refers to nitrogen (N) and phosphorus (P).

1 In non-agricultural systems, internal recycling of both N and P (between plants and soils) typically exceeds total inputs and losses (to or from the atmosphere or waterways) many fold, both globally [44], [101] and at individual sites [102]. Consequently, litter decomposition is much greater than litter loss (*δ_i_L_i_ >> h_i_L_i_*), plant uptake is much greater than losses of plant-available nutrients (*Bν_i_A_i_ >> k_i_A_i_*), SOM mineralization is much greater than SOM loss (*m_i_D_i_ >> φ_i_D_i_*), and SOM mineralization is much greater than the balance of abiotic inputs and plant-available losses (*m_i_D_i_ >>* |*I_i_ – k_i_A_i,i_*|). Exceptions to this pattern will occur where losses are very high (e.g., when ground fires or heavy erosion frequently remove substantial amounts of litter, it is unlikely that *δ_i_ >> h_i_*), where plant or SOM pools are very small (e.g., at the beginning of primary succession, it is unlikely that *m_i_D_i_ >>* |*I_i_ – k_i_A_i,i_*|; see text and analysis), or in heavily polluted regions.

2 See ref [51].

3 Plants typically have an order of magnitude higher N content than P content [44], [103].

4 P is more readily cleaved from organic matter than N (see text).

5 For decomposition, we have defined the parameters such that *ε* controls the relative mineralization of N versus P and *δ* is the overall litter decomposition rate.

6 N and P loss rates via leaching and erosion should be similar because they come from the same organic material. In ecosystems where fire is important, organic N loss rates may be relatively higher than P.

## Results

### Goodness of Fit for Timescale Approximation

**Figure 2:**
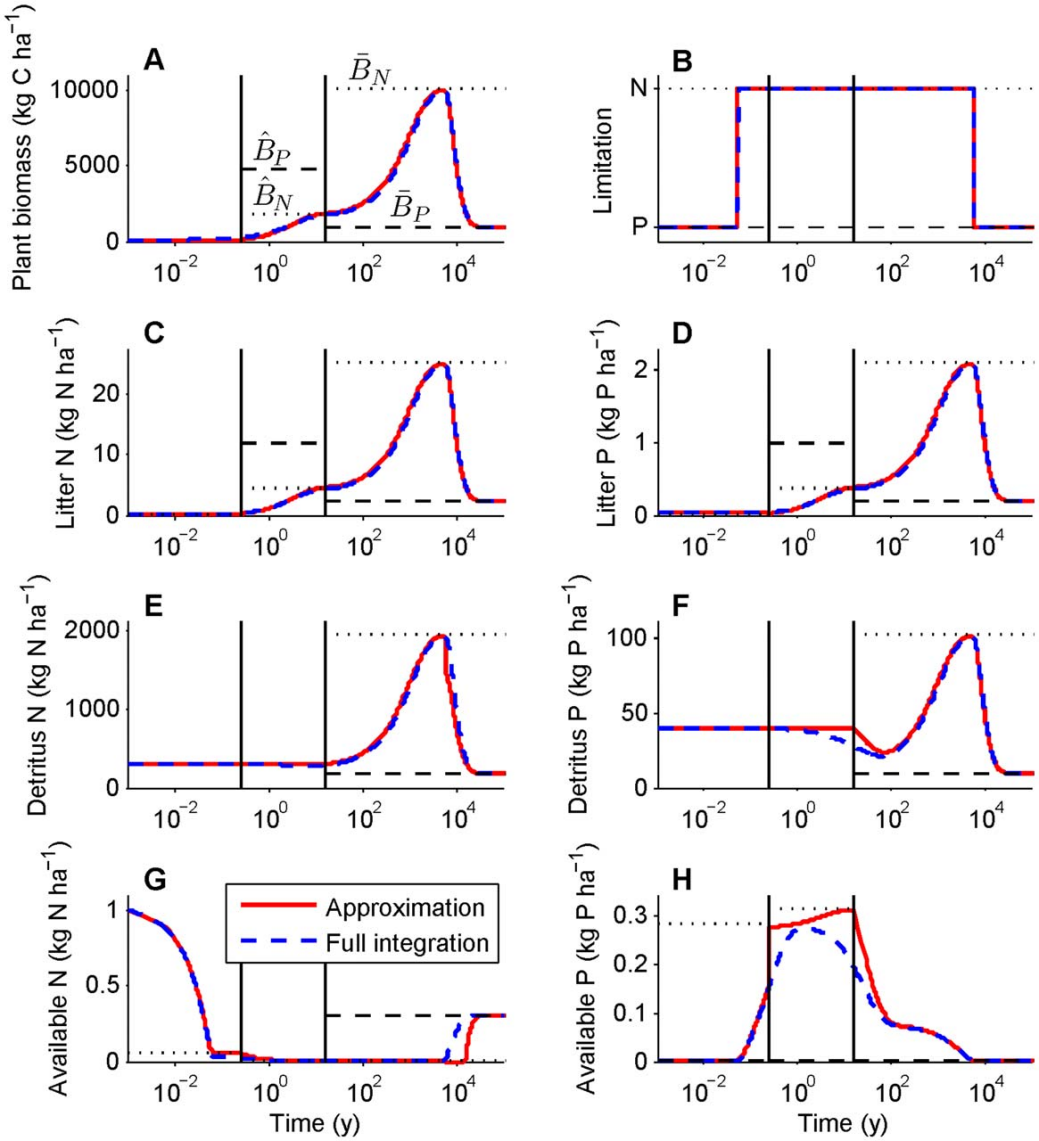
Fit of timescale approximation to full numerical integration. Parameters are as in Table 1, with no symbiotic N fixation. Both the approximation (solid red line) and the numerical integration (dashed blue line) began with biomass  = 300 kg C ha^−1^, litter N and P = 0.1 kg N ha^−1^ and 0.05 kg P ha^−1^, soil organic matter (SOM) N and P = 300 kg N ha^−1^ and 40 kg P ha^−1^, and plant-available N and P = 1 kg N ha^−1^ and 0.003 kg P ha^−1^. Black dotted (N limited) and dashed (P limited) lines are quasi equilibrium and equilibrium values, displayed only for the relevant timescales. Vertical black lines indicate the timescale breaks: we used short timescale approximations to the left of the first black vertical line, intermediate timescale approximations between the two lines, and long timescale approximations to the right of the second line. Panels show (A) plant biomass, (B) which nutrient is limiting, (C) litter N, (D) litter P, (E) SOM N, (F) SOM P, (G) plant-available N, and (H) plant-available P. In this case net primary production begins P limited because there is an abundance of plant-available N, as from a small N fertilization, but becomes N limited within a couple weeks due to the large P:N ratio of inputs relative to plant demand and preferential P recycling. Plants remain N limited through succession and for thousands of years due to the preferential recycling of P and concomitant high loss ratio of dissolved organic N:P. At the long timescale plants become P limited because weathering inputs of P are negligible and dust P inputs are small. The time at which P limitation appears is controlled by the rock P weathering rate. See Fig. 4 for a different development trajectory. Note the logarithmic time axis.

Our timescale approximations fit the full numerical integration extremely well for biomass (Fig. 2A), determining which nutrient is limiting (Fig. 2B), litter N and P (Fig. 2C–D), and the dynamics of the limiting nutrient in SOM and the plant-available pool (Fig. 2E,G when N is limiting, Fig. 2F,H when P is limiting). They fit less well for the non-limiting nutrient in SOM and the plant-available pool (Fig. 2E–H), so we do not put much emphasis on results for the non-limiting nutrient. Explanations for why the deviations occur are in Appendix S1.

### Short Timescale

Our primary questions in this work concern successional timescales (years-centuries) and ecosystem development timescales (millennia and longer), so the short timescale dynamics (hours-years) are less relevant than the intermediate and long timescale results. In the interest of space, we present the short timescale results in Appendix S1 except for the following essential points. All short timescale dynamics converge to globally stable quasi equilibria. For everything but the non-limiting plant-available nutrient, all variables approach their quasi equilibria at the timescale of litter decomposition (a few months for our parameterization). Within the short timescale of our analysis, the limiting plant-available nutrient has two distinct timescales, with the shorter timescale controlled by plant uptake and the longer by litter decomposition. Therefore, the limiting plant-available nutrient quasi-equilibrates with litter before they both quasi-equilibrate at the end of the short timescale (Fig. S1B).

### Intermediate Timescale

To solve for the plant dynamics on the intermediate timescale, we substitute the short timescale quasi equilibrium expressions for litter and plant-available N and P (Appendix S1) for the variables *L_i_* and *A_i_* and assume that SOM (*D_i_*) and weathering P inputs (*I_P_*) are constant. Importantly, these assumptions do not mean that the total amount of organic N and P in soil is constant on the intermediate timescale because litter N and P (*L_i_*) change with plant biomass. This leaves a single differential equation (d*B*/d*t*) and a single variable (plant biomass, *B*) that can be N limited or P limited. Solving for both limitation cases will reveal which nutrient is limiting. If N is limiting, the quasi equilibrium of plant biomass (from setting equation 1 to zero) is


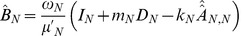
(8)


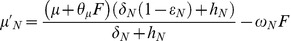
(9)

The double hat indicates the intermediate timescale quasi equilibrium of *A_N_* at the point where *B_N_* quasi equilibrates. Equation 8 can be interpreted approximately as the late successional biomass for a given stage in long-term soil development. Equation 9, which is roughly the turnover rate of plants, defines the controlling rate of the intermediate timescale (see below). A few simplifications will aid the interpretation of these results. First, N limitation is unlikely if N fixers are present in late succession, and they are absent from many late successional forests [68], so we examine the case of no SNF (*F* = 0). Second, mineralization fluxes from SOM far exceed the net balance of inorganic inputs and losses in unpolluted, late successional forests (see Table 2 notes), so we assume that 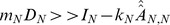. Using these and the assumption that litter loss is small relative to litter decomposition and transfer to SOM (*δ_N_*, *δ_N_*(1−*ε_N_*) >> *h_N_*) (see Table 2),


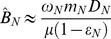
(10)

This means that the main controls on N-limited plant biomass late in succession are net N mineralization (*m_N_D_N_* is net N mineralization from SOM, *ε_N_* is the fraction of litter that is mineralized), the plant’s N use efficiency (*ω_N_*), and the amount of time N is retained within the plant (1/μ).

After solving for the plant biomass quasi equilibrium, the next step is to solve for the transient dynamics by integrating equation 1. Assuming that N-limited plants take up far more plant-available N than is lost (*Bν_N_* >> *k_N_*), the transient dynamics of plant biomass are


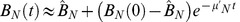
(11)

**Figure 3:**
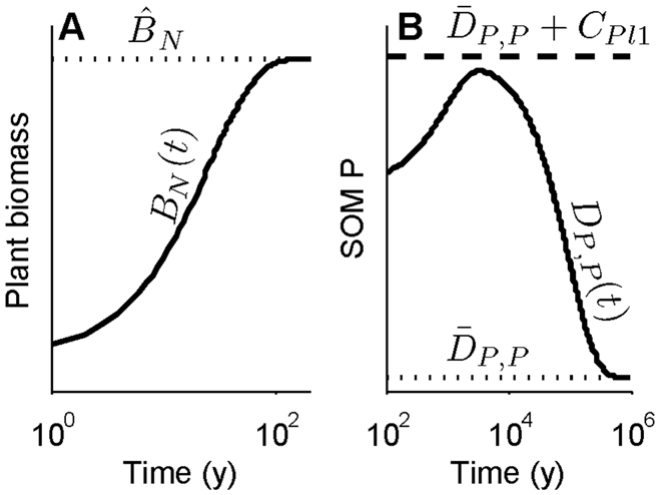
Examples of transient dynamics with single or double saturation rates. (A) On the intermediate timescale, N-limited plant biomass (solid curve) approaches its quasi equilibrium (dotted line) at the rate *µ_N_′* (equation 9, which is the exponent in equation 11). With a logarithmic time axis (as here) this appears sigmoidal, but with a linear time axis it would be a saturating curve (similar to the Michaelis-Menten, or Type II, curve). P-limited plant biomass would have the same shape. (B) When NPP is P limited on the long timescale, SOM P has two controlling rates, the SOM P loss rate (*m_P_*(1−*κ_δP_*) + *φ_P_ ≈ φ_P_*) and the P weathering rate (*ψ*, equation 17). When these are sufficiently different, SOM P (solid line) first approaches an intermediate quasi equilibrium (dashed line) at the SOM P loss rate, then proceeds to its equilibrium (dotted line) at the P weathering rate. Here the rates are different enough to yield an overshoot, but not sufficiently different to yield full quasi equilibration at the intermediate point. Other details of timescale dynamics are given in Figure S1.

**Figure 4:**
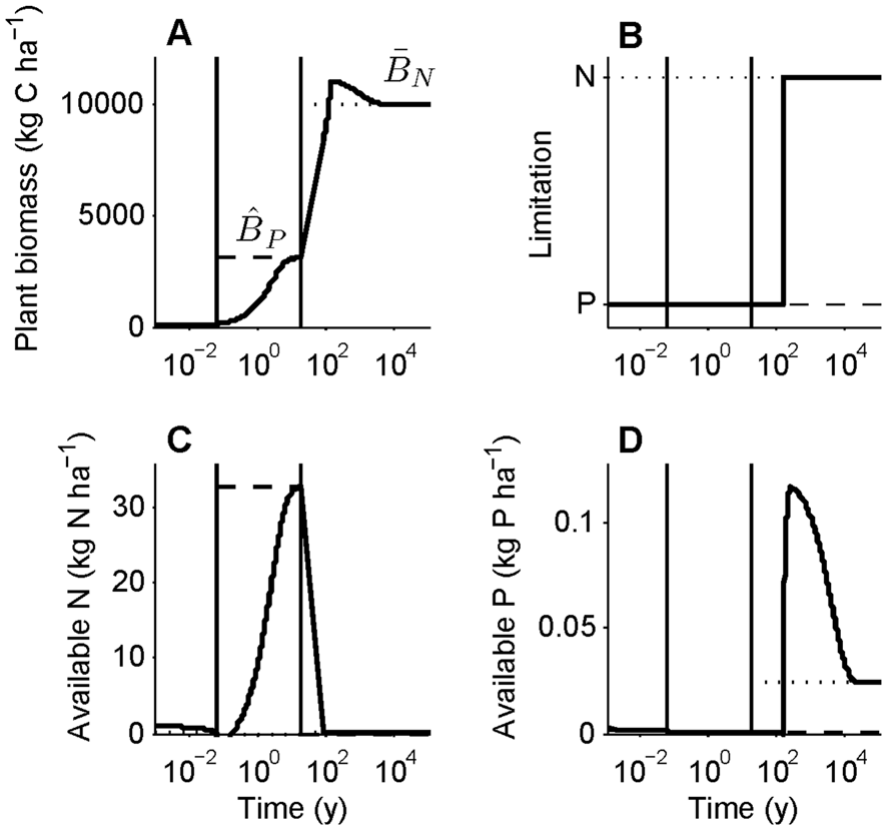
Timescale approximation illustrating the effects of obligate N fixation and high continual P inputs. Parameters and starting conditions are as in Fig. 2 except that *F* = 0.07 kg N kg C^−1^ y^−1^ for the short and intermediate timescales and *α* = 0.2 kg P ha^−1^ y^−1^ throughout. At the end of the intermediate timescale *F* is set to 0 to simulate the exclusion of N fixers. (A) Plant biomass, (B) which nutrient limits net primary production (NPP), (C) plant-available N, and (D) plant-available P are shown. Breaks in timescales, quasi equilibria, and equilibria are shown as in Fig. 2. In this case obligate N fixers fix enough to overcome N limitation, hence P limits NPP through the short and intermediate timescales. Although P weathering depletes rock P as in Fig. 2, relatively high dust deposition and high losses of plant-unavailable N relative to P combine to make N limit NPP at the terminal steady state.

If SNF is below a threshold described in the following paragraph, *µ_N_′* (equation 9) is positive, so N-limited plant biomass begins at *B_N_*(0) and saturates at its quasi equilibrium (Fig. 3A). The saturation rate–how quickly plant biomass approaches its quasi equilibrium–is given by *µ_N_′*; equivalently, the intermediate timescale is 1/*µ_N_′*. With no SNF, the intermediate timescale is approximately 1/*µ*(1−*ε_N_*), the turnover time of plant biomass divided by the proportion of litter decomposition that enters the SOM pool (see above and Table 2). Alternatively, when SNF exceeds the threshold, plants can escape N limitation (i.e., stability is controlled by the sign of *µ_N_′*). This is visible in equation 11 in that *µ_N_′* (equation 9) becomes negative if SNF is sufficiently large, leading to exponential growth of N-limited plant biomass and an unstable quasi equilibrium. In reality this would mean plant growth rapidly becomes P limited, so the N-limited case would become irrelevant, as illustrated in the short and intermediate timescale dynamics in Fig. 4. Fig. 4 differs from Fig. 2 in that obligate N fixers dominate the plant community until the end of the intermediate timescale and dust P inputs are higher.

The threshold that controls stability at the intermediate timescale is when the SNF flux equals the “loss” flux from the litter pool (leaving the system as well as entering the slow SOM pool, which is constant on this timescale). SNF can exceed 100 kg N ha^−1^ yr^−1^ when N fixers are dominant [13], [69], [70], whereas losses from the litter pool–which are less than litterfall inputs–rarely exceed 50 kg N ha^−1^ yr^−1^. Therefore, abundant N fixers have the capacity to overcome N limitation at the intermediate timescale, although they would need to account for the majority of community biomass.

The quasi equilibria and transient dynamics of litter and plant-available N and P can be solved at the intermediate timescale by substituting the plant dynamics (equations 8, 9, and 11) for the plant constant (*B*) in the short timescale quasi equilibria. These are in Appendix S1, with the primary result being that they have the same timescale (*µ_N_′*) and stability as plant biomass.

If P limits NPP at the intermediate timescale, the dynamics of plant biomass, litter N and P, and plant-available P are always stable because there is no plant-controlled P input as there is for N, and the intermediate timescale is approximately 1/*µ*(1−*ε_P_*). The expressions for P-limited quasi equilibria and transient dynamics are identical to the N-limited cases (equations 8–11 and equations in Appendix S1) with the subscript P substituted for N and vice versa, except for slight differences in the equation that describes plant-available N (see Appendix S1).

At the beginning of the intermediate timescale, the plant is N limited if N demand relative to N supply is greater than P demand relative to P supply. Mathematically, this is given by


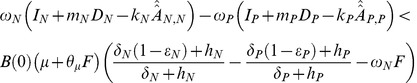
(12)

This is a fairly complicated expression, but the assumptions in Table 2 simplify it substantially, and specific cases of interest are even more easily interpreted. First, consider the case without N fixers (which we examine hereafter in all simplifications about limitation status) during early primary succession. In this case there is virtually no SOM (*D_i_* ≈ 0), and because ecosystem N and P stocks grow rapidly during early succession it is likely that plant-available N and P inputs (*I_i_*) far exceed losses (*k_i_*(*A_i_*)). Using these assumptions, condition 12 simplifies to


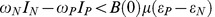
(13)

This shows that the main control on N limitation early in primary succession is the balance of N and P inputs (the dominant controls on supply at this timescale) relative to plant N:P demand. The right-hand side of condition 13 represents the balance of litterfall N versus P that is mineralized before becoming SOM. This term is likely to be positive (*ε_P_*>*ε_N_*) because organic P–generally attached with ester bonds that are easily broken with phosphatase enzymes, which are produced by plants, fungi, and bacteria–is more easily mineralized than organic N [21]. However, this term is likely to be near zero because the overall quantity of litter is low early in succession. Therefore, N versus P limitation in early primary succession depends chiefly on whether the left-hand side of condition 13 is positive or negative. If P inputs (*I_P_*) relative to plant P demand (*ω_P_*) exceed atmospheric N inputs (*I_N_*) relative to plant N demand (*ω_N_*), the left-hand side is negative and N limits NPP.

**Figure 5:**
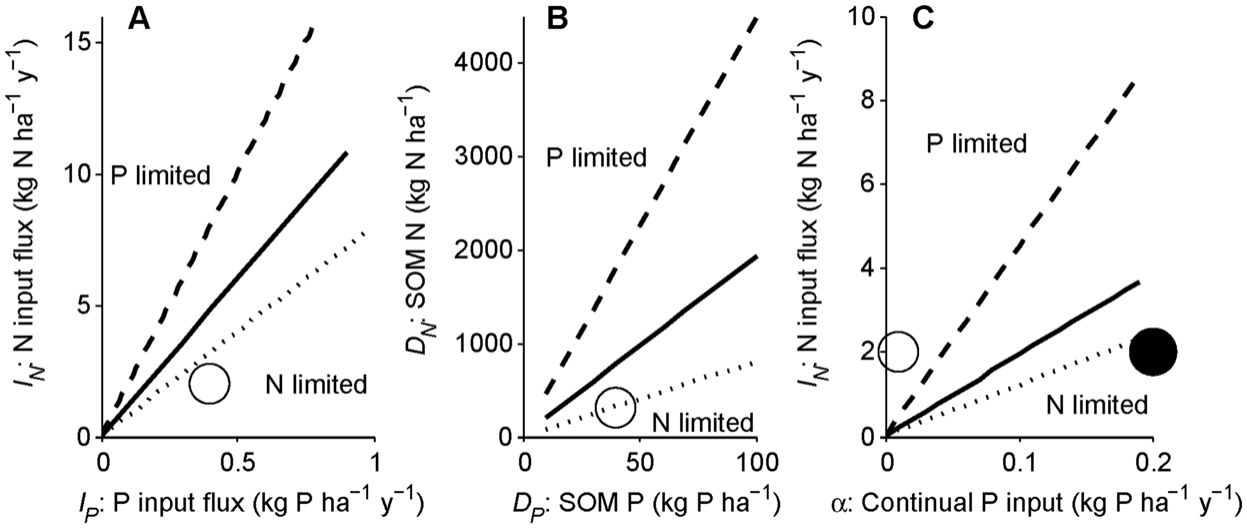
Approximate determination of N versus P limitation at different timescales. (A) The beginning of the intermediate timescale (beginning of succession), (B) end of the intermediate timescale (end of succession), and (C) terminal steady state (end of long-term ecosystem development) are shown with simplifying assumptions from Table 2 and no symbiotic N fixation. (A) At the beginning of succession there is negligible soil organic matter (SOM), so limitation is determined by input fluxes (the axes), plant demand, and litter N versus P mineralization. The dividing line between N and P limitation (condition 13) is plotted with parameters in Table 1 and 10 kg C ha^−1^ initial plant biomass (solid line) as well as for high (litter N:P = 20, P:N mineralization rates = 1.5; dashed line) and low (litter N:P = 8, P:N mineralization rates = 1; dotted line) parameter values. The circle represents parameters in Table 1. Ecosystems with high rock P inputs would be at the right end of the panel. Ecosystems with high N deposition would be near the top of the panel. (B) At the end of succession, mineralization from SOM far exceeds the balance of abiotic inputs and losses in unpolluted ecosystems, so N versus P limitation is determined by SOM N and P (the axes), plant N:P demand, and the mineralization of litter and SOM N versus P (condition 15). The lines are as in (A), and the circle is the initial SOM N and P in Fig. 2. (C) At the terminal steady state, N versus P limitation is determined by the balance of continual inputs and losses, plant N:P demand, and the mineralization of litter and SOM N versus P (condition 18). Axes are N and P input fluxes. Lines and open circle as in (A), with the closed circle indicating the input fluxes for Fig. 4.

Which nutrient is limiting during early primary succession (condition 13) is plotted on Fig. 5A as a function of input fluxes (*I_P_* and *I_N_*). The lines indicate the divide between N and P limitation for a range of plant N:P demand and relative mineralization rates (see Fig. 5 caption). Although our parameter set (open circle in Fig. 5A) yields N limitation, P limitation is plausible in ecosystems with low rock P or slow weathering release of rock P relative to atmospheric N deposition.

The second case we consider is late in primary or secondary succession when plant biomass has quasi equilibrated with soil organic matter, i.e., the end of the intermediate timescale. In this case NPP is N limited at the intermediate quasi equilibrium when


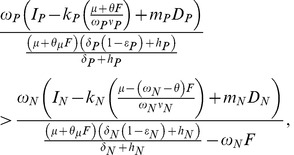
(14)

where *θ* = *θ_µ_*+*θ_g_*. As above, this is equivalent to when N demand relative to supply exceeds P demand relative to supply, but we are interested in going beyond this general statement to determine how each parameter affects the likelihood of N limitation and which are the most important. Decreasing N use efficiency (*ω_N_*), N inputs (*I_N_*), N uptake (*ν_N_*), SNF (*F*), or N mineralization (*δ_N_*, *ε_N_*, *m_N_*, or *D_N_*), or increasing N losses (*k_N_* or *h_N_*) pushes the system toward N limitation, and vice versa with the analogous P parameters. However, a few of these properties are much more relevant than others. Using the simplifying assumptions in Table 2, and again considering the case of no symbiotic N fixers, condition 14 can be simplified to


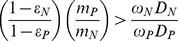
(15)

This shows that limitation at the end of the intermediate timescale is controlled primarily by preferential mineralization of P versus N, the N:P ratio of SOM, and plant N:P demand. The first term in parentheses on the left-hand side of condition 15 is the ratio of litter N to P that remains plant-unavailable during decomposition to SOM. As explained above, the biochemistry of organic matter suggests that P is easier to mineralize than N [21], so the first term is likely greater than 1. The second set of parentheses is also likely greater than 1 for the same reason since it is the ratio of the SOM P:N mineralization rates. The right-hand side of condition 15 is the ratio of SOM N:P relative to plant N:P demand. Plant N:P demand in this model is equivalent to the N:P ratio of litterfall, NUE:PUE, and if δN  =  δP and hN  =  hP (litter decomposition and loss rates are the same for N and P), the N:P ratio of litter. Therefore, the intermediate timescale without SNF ends N limited if P is preferentially mineralized over N by a greater factor than the ratio of SOM N:P to plant N:P demand.

Which nutrient is limiting at the end of the intermediate timescale (condition 15) is plotted on Fig. 5B with the amounts of SOM N and P on the axes, for the parameters in Table 1 (solid line) and a range of litter N:P and N:P mineralization values. The SOM N:P ratio (the x, y coordinate in Fig. 5B) is a strong determinant of N versus P limitation, although the degree of preferential mineralization and the plant N versus P demand (which control the dividing line in Fig. 5B) also play strong roles. The initial conditions for Fig. 2 yield N limitation (open circle in Fig. 5B), although many realistic scenarios would yield P limitation.

**Figure 6:**
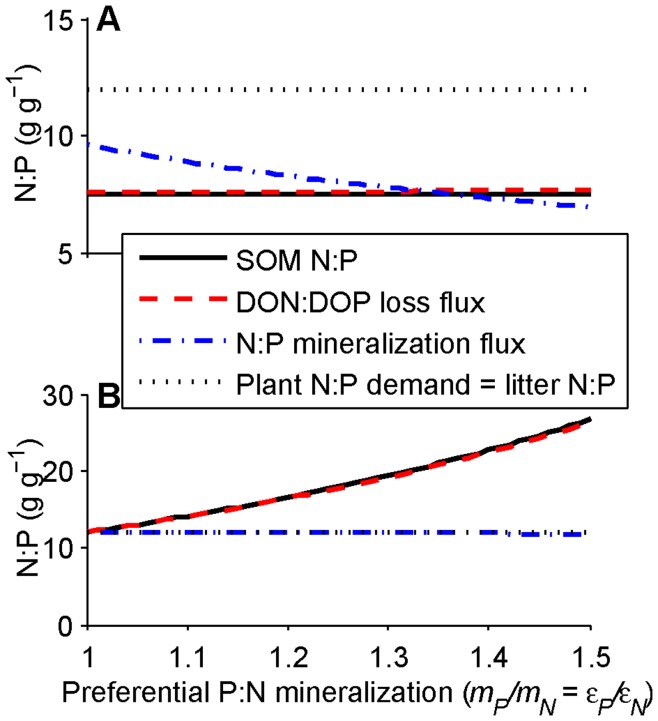
Effects of preferential P versus N mineralization. Effects are different for the (A) intermediate and (B) long timescales. Parameters are from Table 1 except for the mineralization rates *m_N_* and *m_P_* and the fractions of litter N and P decomposition mineralized, *ε_N_* and *ε_P_*, which vary to give the ratios along the horizontal axis. Timescale approximations of soil organic matter (SOM) N:P (solid black line), N:P losses of plant-unavailable nutrients from SOM and litter combined (termed DON:DOP in the legend for brevity, but in reality incorporating dissolved and particulate hydrologic losses and erosion losses; red dashed line), N:P mineralization fluxes from SOM and litter combined (blue dashed-dotted line), and plant N:P demand (black dotted line; which here is equivalent to P use efficiency:N use efficiency, litter N:P, and plant uptake) are plotted. The near-1∶1 correspondence between SOM N:P and DON:DOP stems primarily from the equivalence of *φ_N_* and *φ_P_*, which is not always true (see Table 2 and [37]). However, any monotonic relationship would yield the same timescale dynamics exhibited here.

The effect of preferential mineralization on ecosystem N:P stoichiometry at the end of the intermediate timescale is shown on Fig. 6A, using parameters from Table 1 and initial SOM conditions from Fig. 2. At the intermediate timescale SOM N:P is constant and DON:DOP losses are nearly constant, regardless of preferential mineralization, so increasing P mineralization relative to N causes a decline in the N:P mineralization flux, exacerbating N limitation. If limitation switches during the intermediate timescale, the transition times can be calculated but the expressions are cumbersome, so they are given in Appendix S1.

### Long Timescale

Our long timescale ends in the terminal steady state. The full equilibrium expressions, denoted by bars over the variables, are given in Appendix S1. Even more so than at the intermediate timescale, N limitation at the terminal equilibrium is unlikely if N fixers are present and actively fixing. At the long timescale SNF need only exceed losses of plant-unavailable N to overcome N limitation, as is generally the case in this class of ecosystem models [25]. Except where there are frequent, large fires or erosion, losses of plant-unavailable N are typically well under 10 kg N ha^−1^ yr^−1^ [22]–[24], [71]–[73], whereas fixation fluxes can exceed that by an order of magnitude or more [13], [69].

The long timescale transient dynamics of SOM (*D_i_*) are solved by substituting the intermediate timescale equilibrium expressions into equation 3 and integrating equation 3. Plugging these equations for *D_i_* back into the intermediate timescale quasi equilibrium expressions for plant biomass (equation 11 or its P limitation counterpart), litter, and plant-available nutrients (given in Appendix S1) completes the transient analysis. In the main text we focus on the dynamics of SOM N in the N-limited case and SOM P in the P-limited case because they illustrate the relevant dynamics, but solutions for all variables are presented in Appendix S1.

Transient dynamics of SOM N in the N-limited case and SOM P in the P-limited case are given by


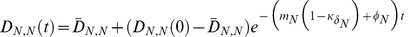
(16)


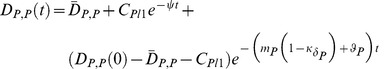
(17)

The new terms *κ_δN_*, *κ_δP_*, and *C_Pl_*_1_ are combinations of parameters, as defined mathematically in Appendix S1. Biologically, at least in the case of no SNF, *κ_δN_* and *κ_δP_* describe the fractions of litter N and P output (decomposition and loss) that remain in the ecosystem. On the long timescale when N limits NPP, the equation describing SOM N dynamics (equation 16) has the same qualitative shape as the equation describing plant biomass dynamics at the intermediate timescale (Fig. 3A): a saturating curve with a single controlling rate (*m_N_*(*1-κ_δN_*) *+ φ_N_*), provided that the rate is positive. Some algebra shows that the rate is positive when losses of plant-unavailable N exceed SNF (see Appendix S1). With no SNF, *κ_δN_* is near 1, so the controlling rate is approximately the SOM N loss rate, *φ_N_*. This is similar to a model that did not explicitly consider litter or SNF, in which the controlling rate of *D_N_* was the SOM N loss rate [26]. On this long timescale plant biomass, litter N and P, and SOM P also have the same ultimate controlling rate as *D_N,N_*(*t*).

When P limits NPP on the long timescale, SOM P (equation 17) has two controlling rates, *m_P_*(1−*κ_δP_*) + *φ_P_* and *ψ*, giving it a qualitatively different shape than SOM N or plant biomass (Fig. 3B). The shape of SOM P depends on the relative values of the controlling rates. The fraction (1−*κ_δP_*) is likely to be so small that the rate *m_P_*(1−*κ_δP_*) is small compared to the loss rate of plant-unavailable P from SOM, *φ_P_*. Therefore, *m_P_*(1−*κ_δP_*) + *φ_P_* is approximately the SOM P loss rate, as was the case for N limitation above.

The second controlling rate, *ψ*, is the rock weathering rate, which is likely to be very slow relative to all other processes discussed in this paper. Assuming *m_P_*(1−*κ_δP_*) + *φ_P_*>> *ψ*, SOM P begins at *D_P_*(0), approaches an intermediate saturation point–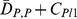
–at the rate *m_P_*(1−*κ_δP_*) + *φ_P_*, then proceeds to its terminal equilibrium at the rate *ψ* (Fig. 3B). Plant biomass, litter N and P, and SOM N share these two slow rates with SOM P. The SOM P buildup over millennia followed by a decline over longer timescales pictured in Fig. 3B is a typical trajectory, but a monotonic decline is also possible if SOM P begins at a high level.

With no or little SNF, and making the assumptions in Table 2, the terminal steady state is N limited when


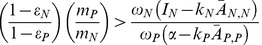
(18)

and P limited if the inequality is reversed.

Condition 18 shows that limitation at the long timescale is controlled primarily by the balance of plant-unavailable N versus P losses and continual inputs of N versus P. The effect of plant-unavailable N versus P losses shows up on the left-hand side of condition 18. The left-hand side of condition 18 is identical to condition 15–the condition for N limitation at the end of the intermediate timescale–and is likely to exceed 1 because of the preferential mineralization of P over N. However, the mechanism behind this effect is different at the long timescale, owing to the feedback between preferential mineralization, soil N and P pools (which are now dynamic), and losses of plant-unavailable N and P. At the intermediate timescale, preferential P mineralization increases the amount of actively cycling P relative to N but does not affect the N:P ratio of SOM because SOM changes so slowly (Fig. 6A). However, at the terminal steady state preferential mineralization of P has no effect on the mineralization fluxes of P versus N since the greater ease of P mineralization is balanced by the increase in SOM N:P that results from preferential P mineralization.

This increase in the N:P ratio in SOM explains the role of plant-unavailable losses in determining N versus P limitation. Because the fluxes of plant-unavailable N and P losses are proportional to the amounts of N and P in SOM, the increase in the N:P ratio of SOM causes an increase in the N:P ratio of DON:DOP losses. This relatively higher loss of plant-unavailable N than P is what can maintain N poor conditions. Fig. 6B shows these long timescale effects using the parameters in Table 1. This increase in DON loss relative to DOP over long timescales suggests a tendency toward N limitation in old soils based on the loss side of the calculation.

The right-hand side of condition 18 illustrates the effects of abiotic N and P inputs on whether N versus P limits NPP at the terminal steady state. When continual P inputs (*α*, e.g., dust deposition and terminal weathering P inputs) are low, the right-hand side of condition 18 is large, suggesting that N limitation is unlikely even if P is preferentially mineralized over N. Alternatively, if dust deposition is high relative to atmospheric N deposition, the long-term equilibrium would more likely be N limited. Which nutrient is limiting at the long timescale (condition 18) is plotted on Fig. 5C with continual P and N input fluxes on the axes, for the parameters in Table 1 (solid line) and a range of litter N:P and mineralization N:P values (dashed, dotted dividing lines). Areas with low dust deposition and low terminal weathering rates are likely to be P limited (e.g., Fig. 2; open circle in Fig. 5C), but ecosystems with larger continual P inputs would more likely be N limited at the terminal steady state (e.g., Fig. 4; closed circle in Fig. 5C) particularly given the greater losses of SOM N than P.

**Figure 7:**
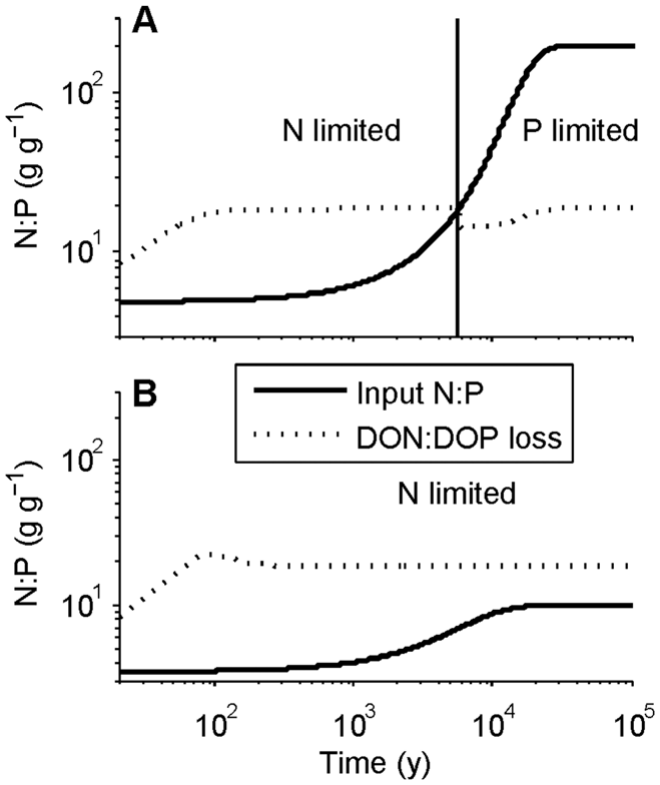
Contrasting effects of inputs versus losses in determining N versus P limitation over long timescales. Effects are shown for simulations in (A) Fig. 2 and (B) Fig. 4. The N:P ratio of plant-unavailable losses (termed DON:DOP in the legend for brevity, but in reality incorporating dissolved and particulate hydrologic losses and erosion losses; dotted lines) increases over decades due to preferential P mineralization (see Fig. 6), increasing the likelihood of N limitation over the intermediate and the beginning of the long timescales. On millennial and longer timescales the input N:P ratio increases due to the decline in P weathering, increasing the likelihood of P limitation over very long timescales. The transition time from N to P limitation in Fig. 2 corresponds to the crossing of the input and loss N:P ratio lines in (A) (indicated by the vertical line), which is controlled primarily by the weathering rate (see Fig. 8). In Fig. 4 and (B) there is no transition to P limitation over long timescales because the input N:P remains lower than the DON:DOP loss ratio, despite increases in both. The condition for whether limitation switches from one nutrient to the other is given in conditions 14, 15 and 18 (also see Fig. 5). The dip in the DON:DOP loss ratio when the input and loss line cross (when limitation switches) in (A) is an artifact of the timescale separation approximation–it remains constant at its saturation level in the full numerical integration (results not shown)–but all other trends reflect the true dynamics of the system. Note that both axes are logarithmic.

The contrasting effects of inputs and plant-unavailable losses on nutrient limitation over the long timescale are summarized in Fig. 7. The N:P ratio of plant-unavailable losses increases at the beginning of the long timescale due to preferential P mineralization, which increases the N:P ratio of SOM and thus the ratio of plant-unavailable DON:DOP losses. This pushes the ecosystem toward or exacerbates N limitation. Over geological timescales the N:P ratio of inputs increases due to declining P inputs from rock weathering, which pushes the ecosystem toward or exacerbates P limitation. Ultimately the balance of input and plant-unavailable loss N:P ratios determine limitation at the terminal steady state (condition 18; Fig. 5). However, the contrast in the timescales of dissolved organic matter leaching (slow but not glacial) and rock weathering (glacial) means ecosystems that are N limited at the end of intermediate timescales will likely remain so at least until well into the geological timescale.

As with the intermediate timescale, there may be a transition from N to P limitation or vice versa at the long timescale. A transition from P to N limitation would be driven by the increase in the ratio of losses of plant-unavailable N:P over the SOM timescale. Conversely, a transition from N to P limitation would be driven by the decrease in P input over rock weathering timescales (Fig. 7). Because the SOM loss timescale is likely to be shorter than the rock weathering timescale, the DON:DOP loss ratio rises faster than the input N:P ratio (Fig. 7), so the transition from N to P limitation is much more likely to occur on the rock weathering timescale (see Appendix S1 for details). If so, the transition time from N to P limitation is


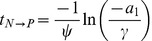
(19)

**Figure 8:**
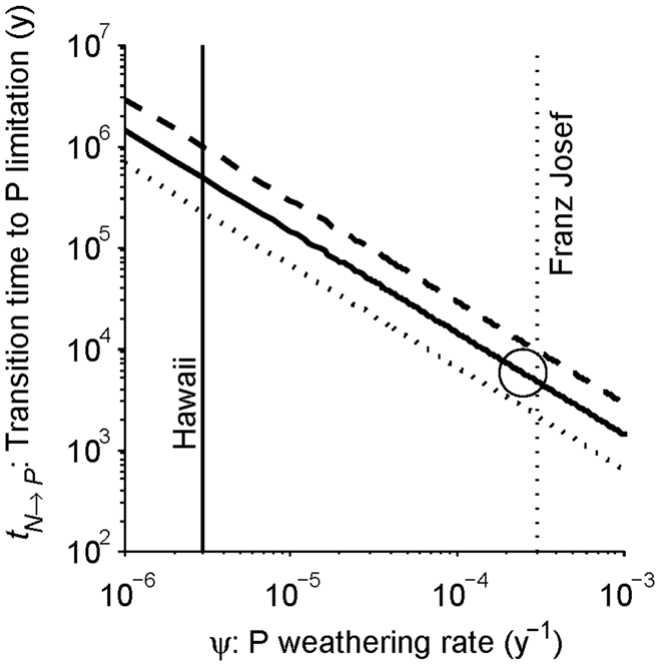
Transition time to P limitation on the weathering timescale. The main determinant of the transition time, if it occurs on the weathering timescale, is the weathering rate. Equation 19 is plotted as a function of the weathering rate, with the rest of the parameters as in Table 1 (solid diagonal line), high dust deposition and parent material P (*α* = 0.05 kg P ha^−1^ y^−1^, *γ* = 1 kg P ha^−1^; dashed diagonal line) and low dust deposition and parent material (*α* = 0.001 kg P ha^−1^ y^−1^, *γ* = 0.2 kg P ha^−1^; dotted diagonal line). The weathering rate and transition time in Table 1 and Fig. 2 are plotted as an open circle. All parameter sets plotted here yield a transition to P limitation, although many possible parameters sets do not (see Figs. 4, 5). Weathering rates from the Hawai’i (solid vertical line) and Franz Josef (dotted vertical line) chronosequences from [51] are also plotted. Note that both axes are logarithmic.

Faster weathering rates (larger *ψ*), lower initial parent material P content (smaller *γ*), and slower dust deposition fluxes (smaller *α*, which means smaller *a*_1_; a combination of constants defined in Appendix S1) make the transition occur sooner (smaller *t_N→P_*). The primary control over the transition time is the weathering rate since it is the only term outside the logarithm and it varies substantially across ecosystems [51], so the transition time is plotted as a function of the weathering rate in Fig. 8. The solid line represents the parameters in Table 1, whereas the dashed and dotted lines represent a range of parent material P and continual P input fluxes that still yield long-term P limitation. The switch in limitation is highly dependent on the weathering rate, and for these parameters the transition occurs at approximately 1/*ψ* (i.e., at 10^3^ years for a weathering rate of 10^−3^/y, 10^6^ years for *ψ* = 10^−6^/y, and so on).

## Discussion

Our model allows for a diversity of ecosystem development trajectories. One of these is a transition from N to P limitation over long timescales, as observed across the Hawaiian archipelago [4], [37] and inferred in New Zealand [3], [5] and Arizona [6], [7]. In this case, depicted in Fig. 2, very young soils with negligible soil organic matter tend toward N limitation primarily because P weathering inputs relative to plant demand exceed atmospheric N deposition relative to plant demand. As succession proceeds and SOM accumulates over decades to centuries, this input imbalance is compounded by the higher fraction of organic P returned to the plant-available pool than organic N. This recycling imbalance increases the N:P ratio of SOM, and the resulting high N:P ratio of plant-unavailable nutrient losses exacerbates N limitation. However, when P inputs decline to a sufficiently low level over geological timescales the input effect trumps the plant-unavailable loss effect, yielding a terminal P-limited steady state. This case shows that declining P inputs–which are the primary cause of the transition to P limitation–are sufficient to explain declines in biomass, productivity, soil P, and soil N observed during ecosystem retrogression [8]. Other factors could also contribute to these retrogression patterns, as discussed in [8], and must if retrogression occurs when P is not limiting.

Our model also allows for P limitation on young soils and the persistence of N limitation on very old soils. Ecosystems with abundant and active N fixers are unlikely to remain N limited, although co-limitation might be more common than P limitation if SNF is regulated based on demand [40], [74], [75] or if community-level processes are fast enough to balance the abundance of N fixers versus non-fixers. Even without SNF, young ecosystems can be P limited if they have P-poor parent material, low weathering rates (termed “transactional” P limitation by [76]), or high N inputs. In these cases, however, a transition from P to N limitation during succession is possible if N fixers are excluded during succession or if preferential recycling of P over N is sufficiently large. On old soils with low or no SNF, ecosystems with high dust P deposition relative to atmospheric N inputs are likely to remain N limited indefinitely, particularly given that losses of plant-unavailable N are high relative to losses of plant-unavailable P. Fig. 4 illustrates one of these cases, where obligate N fixers overcome N limitation early in succession, but high dust deposition and the exclusion of symbiotic N fixers yields an N-limited terminal steady state.

A common feature of these trajectories is that regardless of their initial limitation status, ecosystems should experience increasing N deficiency relative to P over decades to millennia but increased P deficiency relative to N over geologic timescales. At decadal timescales the shift toward N limitation occurs because of increased P mineralization, which directly alleviates P limitation, and possibly because of a loss of N fixers from the community. At centurial to millennial timescales the shift toward N limitation occurs because of greater losses of SOM N relative to SOM P–which itself results from preferential P mineralization–and possibly due to a loss of N fixers as well. The shift toward P limitation over geologic timescales derives from declining P inputs, as in the classic argument of Walker & Syers [3]. These contrasting effects at different timescales stem from well-known biogeochemical mechanisms, yet are typically not discussed together in the context of ecosystem development.

### Symbiotic N Fixation Overcomes N Limitation

As noted elsewhere [21], [25], [39], [53], [75], [77], SNF has the capacity to overcome N limitation if N fixers are abundant and active. Our model agrees with this notion, and as with the models in [25], [39], specifies that SNF need only exceed plant-unavailable “losses” to overcome N limitation if SNF is proportional to plant biomass. Unlike previous work, however, the model in the present work shows that the exact definition of plant-unavailable losses changes at different timescales. At the terminal equilibrium plant-unavailable “losses” are losses from the entire ecosystem such as leaching of DON. Alternatively, on the plant timescale plant-unavailable N “losses” include the flux of litter to SOM because SOM turns over so slowly that litter decomposed to recalcitrant SOM is effectively lost for decades. Strong plant-unavailable N loss vectors, such as frequent fires, may be able to maintain N limitation in the face of SNF, but except for these cases SNF can likely overcome N limitation at each timescale if N fixer abundance and activity are not constrained.

Because of this capacity to overcome N limitation, understanding N fixer abundance and activity is paramount to understanding N versus P limitation at all timescales, and has been the subject of much attention. Symbiotic N fixers are abundant in some early successional temperate forests [5], [9], [10], [68], and there is evidence that these species–such as *Alnus rubra* [13], [71], [73] and *Coriaria arborea* [78]–are highly active N fixers regardless of soil N availability (i.e., they are functionally obligate). In such cases N would likely not limit these N fixers, so limitation by P or some other factor to SNF might be common [15], [21], [53], [77]. In addition to limitation to SNF, P limitation to NPP might be common in early successional temperate forests dominated by active N fixers. This P limitation would seem paradoxical both because N fixers likely would not have established without N limitation and because N fixation beyond the point of co-limitation would appear energetically wasteful [24], [75].

Despite these seeming paradoxes, observations of high plant-available N losses from forests co-dominated by alder in the Pacific Northwest of the United States [13], [71], [73] and high N availability after 60 years of *Coriaria arborea* dominance along the Franz Josef chronosequence in New Zealand [5] are consistent with–though not direct evidence of–P limitation, as our Fig. 4B,C shows. Obligate SNF might be due to high costs of being facultative or time lags in regulating SNF [40], although empirical tests of these hypotheses are currently lacking. As succession proceeds and non-fixers outcompete fixers, there can be a shift toward co-limitation and ultimately to N limitation on the intermediate timescale because much plant-available N is lost and preferential P recycling takes its toll. Long-term fertilization studies that track SNF and NPP simultaneously would help bolster these interpretations.

Although many young temperate forests are dominated by symbiotic N fixers, many have none [9], [68], and symbiotic N fixers are virtually absent from old temperate forests [21], [68]. Understanding N fixer rarity is equally important in the search to understand N versus P limitation in temperate forests. P limitation to SNF–which has been shown experimentally in Alaskan forests [15]–might explain N fixer rarity [21], [53], as might high energetic costs of SNF [21], [79], limitation of SNF by another nutrient such as molybdenum [21], preferential herbivory on symbiotic N fixers [21], [39], [53], [80]–[82], low nitrogen use efficiency in N fixers [39], or a tradeoff between SNF and soil N uptake [39], [79]. It is unlikely, however, that a phylogenetic constraint explains N fixer rarity in late successional temperate forests, at least in the United States [68].

Unlike temperate forests, many tropical forests seem to have abundant N fixers [69], [75], [83], although there are notable exceptions [37], [75]. N limitation is therefore unlikely in tropical forests with abundant N fixers, regardless of their soil age, although because tropical N fixers seem to adjust SNF based on soil N [74] and/or P [84] content, co-limitation by N and P might be common [40], [75].

### Successional and Ecosystem Development Trajectories without Symbiotic N Fixation

**Figure 9:**
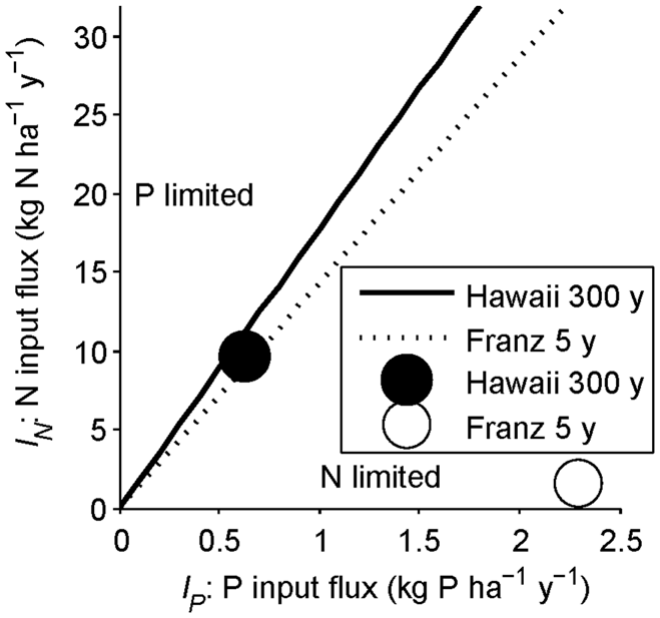
Model predictions for N vs. P limitation in the youngest Hawai’i and Franz Josef sites. Axes and equations are as in Fig. 5, but with Hawai’i in a solid line and closed circle and Franz Josef in a dotted line and open circle. At the beginning of primary succession the Hawai’i site would be N limited but fairly close to co-limitation, whereas the Franz Josef site would be strongly N limited without any symbiotic N fixation. Input and nutrient use efficiency parameters come from the 300 year old Hawaiian site (*I_N_* = 9.6 kg N ha^−1^ y^−1^, *I_P_* = 0.63 kg P ha^−1^ y^−1^, *ω_N_ = *382 g C g N^−1^, *ω_P_* = 6780 g C g P^−1^ [37]) and the 5–7 year old New Zealand site (*I_N_* = 1.5 kg N ha^−1^ y^−1^ [78], *I_P_* = 2.2 kg P ha^−1^ y^−1^–calculated from the decrease in soil inorganic P from the 5 to the 60 year old site–*ω_N_ = *45 g C g N^−1^, *ω_P_* = 643 g C g P^−1^ [5]), with the other parameters as in Fig. 5a.

Even without SNF, it is conceivable that many young ecosystems could be P limited due to high N deposition or low P inputs, particularly as atmospheric N deposition rises with anthropogenic activity [32], [76]. In light of these input effects, we examined how well our model predictions matched observations of young forests in Hawai’i and Franz Josef. The available data suggest that the 300 year old Hawaiian site has relatively high abiotic N inputs (owing in part to volcanic N fixation) and moderate abiotic P inputs (owing mostly to weathering) [37]. Plotting these N and P inputs on our early succession figure suggests that NPP in young Hawaiian forests is N limited, but not far from co-limitation (Fig. 9; solid line and closed circle). This prediction of N-limited plant growth is consistent with fertilization experiments in the 300 year old site and a nearby 26 year old site [85]. The 26 year old site is a much better match for the assumptions in Fig. 9 (chiefly, negligible SOM). However, our model suggests that N limitation should become stronger through 300 years due to preferential P mineralization, so the prediction of N-limited NPP early in primary succession is consistent with N-limited NPP in the 300 year old site. The Franz Josef sites have relatively low abiotic N inputs and high estimated abiotic P inputs (see Fig. 9 caption), so our model indicates that NPP in the youngest Franz Josef site (5 years old, which has negligible SOM) would be strongly N limited without SNF (Fig. 9; dotted line and open circle). Although there have been no fertilization experiments at Franz Josef, this prediction matches inferences based on soil nutrients and the dominance of symbiotic N fixers [5].

Without SNF, preferential recycling of P [21] is the dominant factor that favors N limitation at the intermediate timescale (i.e., the end of primary succession on young soils), although substantial N inputs from SNF or anthropogenic emissions could tip the scale toward P limitation. At the beginning of the long timescale, preferential P mineralization increases the SOM N:P ratio and consequently the N:P ratio of plant-unavailable losses, which exacerbates N limitation long before weathering P inputs decline. This highlights one of the counterintuitive results from our analysis: an increase in the soil N:P ratio over time should not be taken as an indication of N sufficiency or P limitation. Increasing soil N:P over time can stem from increased return of P to plants (over decadal to timescales) and larger losses of plant-uncontrollable N relative to P (over centurial timescales), both of which indicate increasing N deficiency.

These soil dynamics are observed at Franz Josef, where the ratio of N to organic P in the top 10 cm of mineral soil rises from less than 1 (5 year old site) to 3 (60 years) to 7 (130 years) to 12 (280 years) to 17 (500 years), then remains at 15–17 until 60,000 years before jumping to 34 at 120,000 years [5], [45]. Mineral soil N to organic P does not rise substantially over time in Hawaii (27 at 0.3 ky, then 7–13 from 2.1–4100 ky in the top 50 cm [86]) or Arizona (25 at 1 ky, 22 at 55 ky, 30 at 750 ky, 17 at 3000 ky in the top 15 cm [6], [7]), although both of these sequences start at an age when soil N:P may already have equilibrated (centuries to a millennium).

At the end of ecosystem development, nutrient limitation is determined by the balance of continual input (such as atmospheric N and dust P deposition) and loss fluxes. Although the Hawaiian chronosequence experiences little dust P deposition, even that small amount is biologically relevant [17], and a large fraction of the globe receives much greater dust P deposits. Large parts of Africa, Eurasia, and Australia and some parts of South America and the Caribbean receive between 0.1 and 1 kg dust P ha^−1^ y^−1^ [18], which according to our model could maintain P sufficiency indefinitely (Figs. 4, 5C). A careful analysis of global projections of N and P deposition [18], [30] combined with plant-unavailable N versus P loss data could reveal which areas of the globe are likely to be N limited versus P limited at steady state. For example, parts of southern Chile and Argentina have substantial dust P deposition [18], low N deposition [30], and substantial DON losses [22], [23], suggesting N limitation. Consistent with this, some old soils in Chile and Argentina display low plant-available N losses [22], [23] and substantial retention of added N [65], [87]. Replenishment of parent material P by tectonic uplift can also be substantial across much of the globe [19], strengthening the argument that a terminal N-limited steady state is plausible in many ecosystems.

If limitation transitions from N to P at the long timescale, our model suggests that the P weathering rate (with units of [time^−1^]) is the dominant control over when the transition occurs, although properties such as the initial parent material P and dust P deposition also play roles. Weathering rates from Franz Josef (dotted vertical line) and Hawai’i (solid vertical line) estimated by [51] are plotted on Fig. 8 to illustrate these transition times. Based on these rate estimates and our model, the transition occurs sooner in Franz Josef than in Hawai’i–matching predictions from Richardson et al. [5]–somewhere between 1000 and 10,000 years. In the case of Hawai’i, our model predicts that the transition occurs between 200,000 and 1 million years, which is later than observed given the results showing co-limitation [4] at 20,000 years. However, the weathering rate is the only parameter fit to these sites on Fig. 8–all other parameters to make the transition time curve come from our generic set in Table 1–and uncertainty in the weathering rates would substantially alter these predictions.

### Model Omissions and Opportunities for Future Development

As in any model, we have omitted many features of reality. Perhaps the most interesting and important of these concerns biotic responses to nutrient limitation. Theory [88]–[90] and data [91] suggest that organisms often respond to imbalances in nutrient supply versus demand. These responses can occur over physiological (plastic) or evolutionary (genetic) timescales, and tend to make organisms approach co-limitation in many models [40], [92]–[97]. However, plasticity constraints [98], [99], time lags in plastic and/or evolutionary change [100], or other constraints [39] can prevent nutrient co-limitation in models [25], [40], [95]. Therefore, unlike co-limitation between a nutrient and light (which is absent about half of each day) or water (which is highly variable over short timescales), co-limitation between multiple nutrients might not be ubiquitous.

For simplicity, our model does not include biotic adjustments to counteract limitation by a single nutrient; it stipulates that NPP is limited by either N or P except at the exact ratio that yields co-limitation. Previous theoretical work [38], [40], [79], [95]–[97] shows that such biotic adjustments can yield co-limitation under some or all environmental conditions, depending on how they are incorporated into the model. This past work also shows that the approach to co-limitation may be very rapid, intermediate, or slow. Because such adjustments are likely to occur in nature, our statements about N versus P limitation should be taken as tendencies toward certain limitations, with the magnitude of the tendencies proportional to the distance from co-limitation.

### Conclusion

In the present work we offer a simple, quantitative set of predictions for whether N or P is more likely to limit NPP at different stages in ecosystem development. Most of the qualitative predictions are in line with intuition. For example, increasing P inputs and preferential mineralization and retention of P decrease the likelihood of P limitation. However, our theoretical analysis goes beyond this intuition, specifying how strong these effects need to be to push the ecosystem from one state to another and the exact timescales at which they are important. From a theoretical standpoint, our analytical solutions of transient dynamics offer a framework to address myriad questions–such as how environmental factors influence N versus P limitation–in a more interpretable and rapid way than relying on simulations alone.

One counterintuitive result revealed by this analysis concerns the implications of changing N:P ratios in soil organic matter. Although rising SOM N:P seems like it might indicate increasing N sufficiency, our analysis shows that the opposite could be true. Preferential P mineralization acts to transfer P from SOM to the biota, and the resulting increase in SOM N:P yields greater loss of organic N relative to P, which in turn promotes N deficiency. This prediction could be supported empirically with data showing increasing N relative to P limitation (from fertilization experiments) as SOM N:P rises. Additionally, data showing a positive, monotonic relationship between SOM N:P and the N:P ratio of losses of plant-unavailable nutrients (the combination of erosion and dissolved and particulate hydrologic losses) would support the mechanism behind this result. A negative, monotonic relationship would refute this long timescale mechanism of increasing N limitation, whereas a non-monotonic relationship would suggest that the mechanism holds under some conditions but not others.

One of the most empirically useful contributions of this work might be that surprisingly few quantities are needed to answer certain questions. For example, knowledge of continual inputs, losses of plant-available nutrients, nutrient use efficiencies, and the degree of preferential mineralization of P versus N are sufficient to determine whether N or P is more likely to limit NPP at the terminal steady state (condition 18). Because most of our analytical results depend heavily on timescale separation, time series (for short or intermediate timescales) or chronosequence (for intermediate or long timescales) data exhibiting multiple saturations of the variables included here (as in Figs. 2, 3, 4 and Fig. S1, with time on a log scale) would provide strong support for these assumptions. More generally, the greatest empirical needs for evaluating this model are more quantitative data on P weathering and dust inputs and preferential P mineralization (for parameterization), and crucially, more fertilization studies testing for N versus P limitation in forest ecosystems on old soils around the world.

## Supporting Information

Figure S1 **Examples of transient dynamics at the short timescale.** (A) Litter N approaches its quasi equilibrium in a saturating manner with a rate controlled by the exponent in equation S2 in Appendix S1. Litter P would be qualitatively similar. Depending on whether the perturbation increases (e.g., a storm that blows leaves down) or decreases (e.g., a ground fire) litter stocks, the saturation will approach from above or below. Short timescale litter dynamics do not depend on which nutrient limits plant growth. (B) Limiting plant-available nutrient dynamics if limitation does not switch on the short timescale. If plant uptake is faster than litter decomposition there are two separate saturations, which can take a variety of shapes (solid lines give two examples; see Appendix S1 for details).
(PDF)

Figure S2 **Simulation to evaluate effects of forced rapid fluctuations.** Pools and fluxes of plant-available nutrients fluctuate rapidly due to changes in soil moisture, among other things, which would mean that they would fluctuate around the quasi equilibria we present in this ms. We conducted additional simulations to evaluate whether these rapid fluctuations would propagate up to longer timescales. These simulations used the same conditions as in Figure 2 except that each of the soil (*δ_i_*, *h_i_*, *m_i_*, *φ_i_*, and *k_i_*) and/or plant (*ν_i_*, *F*) rate parameters vary as sine functions of time, with 10–100 fluctuations per year. The parameters varied ±50–90% of their base values. Specifics for the run shown here were that soil parameters only (not plant parameters) varied 20 fluctuations per year and ±90% variation for each parameter, and the simulation was run for long enough to evaluate the fit at the long timescale (8000 y). This combination exhibited among the largest discrepancies from the results in Fig. 2 of any of the values we tried, which show up in the very short timescale for plant-available N and P. However, it is still very close to the original results, particularly for the longer timescale variables, leading us to conclude that rapid fluctuations such as these would not strongly affect our results.
(PDF)

Appendix S1 **Derivations and additional results.**
(PDF)
