# Supplementary figures and images for: Nitrogen and Phosphorus Limitation over Long-Term Ecosystem Development in Terrestrial Ecosystems
Source: PLoS One. 2012 Aug 3;7(8):e42045. doi: 10.1371/journal.pone.0042045 (PMC3411694; doi:10.1371/journal.pone.0042045)

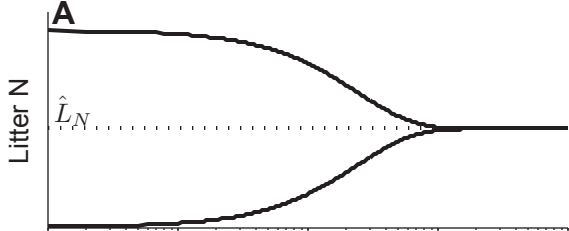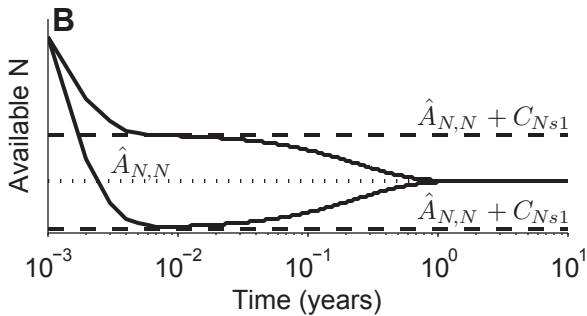

Supplement: Figure S1 — Examples of transient dynamics at the short timescale. (A) Litter N approaches its quasi equilibrium in a saturating manner with a rate controlled by the exponent in equation S2 in Appendix S1. Litter P would be qualitatively similar. Depending on whether the perturbation increases (e.g., a storm that blows leaves down) or decreases (e.g., a ground fire) litter stocks, the saturation will approach from above or below. Short timescale litter dynamics do not depend on which nutrient limits plant growth. (B) Limiting plant-available nutrient dynamics if limitation does not switch on the short timescale. If plant uptake is faster than litter decomposition there are two separate saturations, which can take a variety of shapes (solid lines give two examples; see Appendix S1 for details). (PDF) [file pone.0042045.s001.pdf]

Plant biomass (kg C ha<sup>-1</sup>)

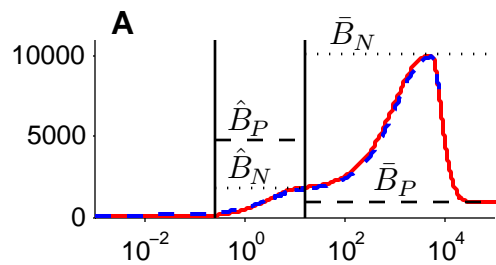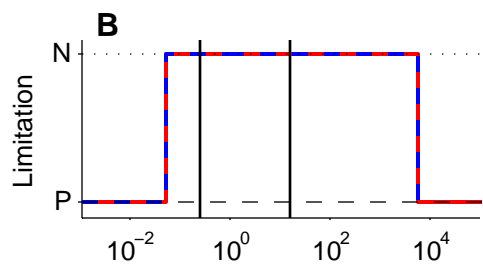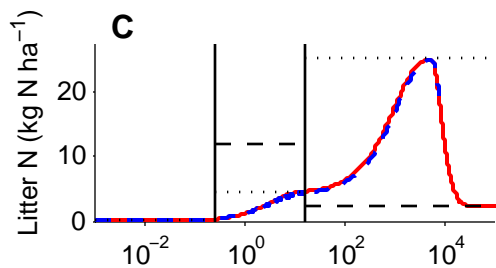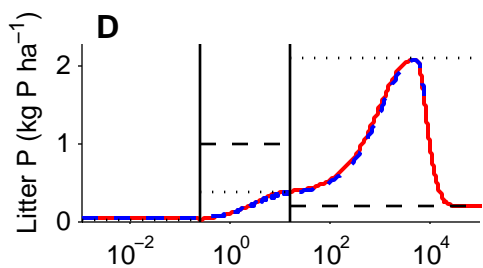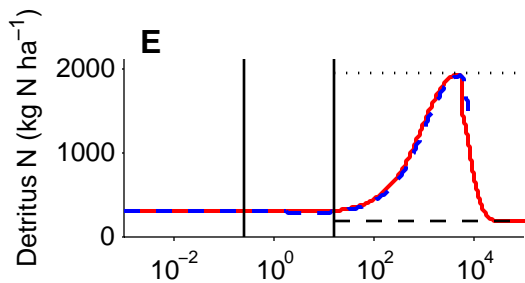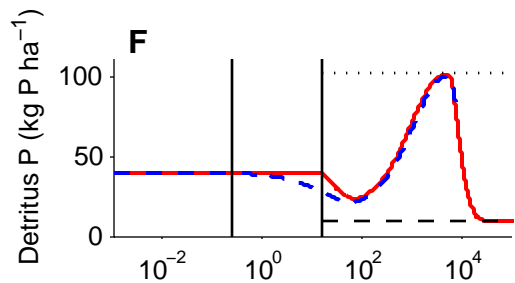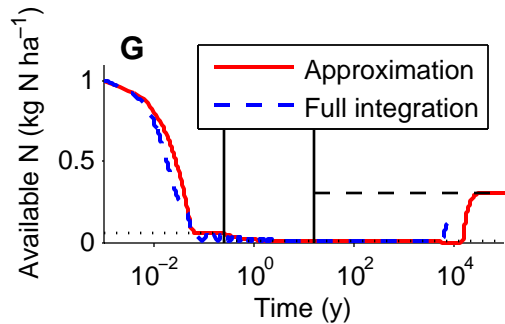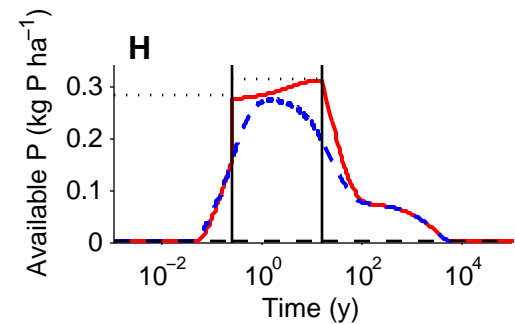

Supplement: Figure S2 — Simulation to evaluate effects of forced rapid fluctuations. Pools and fluxes of plant-available nutrients fluctuate rapidly due to changes in soil moisture, among other things, which would mean that they would fluctuate around the quasi equilibria we present in this ms. We conducted additional simulations to evaluate whether these rapid fluctuations would propagate up to longer timescales. These simulations used the same conditions as in Figure 2 except that each of the soil (δi, hi, mi, φi, and ki) and/or plant (νi, F) rate parameters vary as sine functions of time, with 10–100 fluctuations per year. The parameters varied ±50–90% of their base values. Specifics for the run shown here were that soil parameters only (not plant parameters) varied 20 fluctuations per year and ±90% variation for each parameter, and the simulation was run for long enough to evaluate the fit at the long timescale (8000 y). This combination exhibited among the largest discrepancies from the results in Fig. 2 of any of the values we tried, which show up in the very short timescale for plant-available N and P. However, it is still very close to the original results, particularly for the longer timescale variables, leading us to conclude that rapid fluctuations such as these would not strongly affect our results. (PDF) [file pone.0042045.s002.pdf]
