# Supplementary material for: Nitrogen and Phosphorus Limitation over Long-Term Ecosystem Development in Terrestrial Ecosystems
Source: PLoS One. 2012 Aug 3;7(8):e42045. doi: 10.1371/journal.pone.0042045 (PMC3411694; doi:10.1371/journal.pone.0042045)
Supplement: Appendix S1 — Derivations and additional results. (PDF) [file pone.0042045.s003.pdf]

Nitrogen and phosphorus limitation over long-term ecosystem development in terrestrial ecosystems

Duncan N. L. Menge[*1,2,3] and Lars O. Hedin[1] and Stephen W. Pacala[1]

[1] Department of Ecology and Evolutionary Biology, Princeton University, Princeton, NJ, USA

[2] National Center for Ecological Analysis and Synthesis, Santa Barbara, CA, USA

[3] Department of Biology, Stanford University, Stanford, CA, USA

* Author to whom correspondence should be addressed: Email: dmenge@princeton.edu

Appendix S1: Derivations and additional results

P input derivation

To a first approximation, uplift and erosion act to replenish weatherable P in the ecosystem independently of the quantity of weatherable P, and weatherable P becomes an input to the ecosystem based on the weathering rate. If W is weatherable P, u is the uplift/erosion flux, and ψ is the weathering rate, the change in weatherable P over time is

$$\frac{dW}{dt} = u - \psi W$$

Thus, the equilibrium amount of weatherable P and the amount of weatherable P at any time t are given by

$$\overline{W} = \frac{u}{\psi}$$

$$W(t) = \overline{W} + \left(W(0) - \overline{W}\right)e^{-\psi t}$$

The P weathering input into the ecosystem is given by $\psi W(t)$, so the total P input is given by dust (d, assumed to independent of ecosystem age) and weathering inputs:

$$I_P(t) = d + \psi W(t)$$

$$I_P(t) = d + u + \left(\psi W(0) - u\right)e^{-\psi t}$$

Therefore, the constant input is composed of dust inputs and the uplift/erosion flux: $\alpha = d + u$, and the initial time-dependent flux is given by $\gamma = (\psi W(0) - u)$.

Why the timescale approximation for non-limiting nutrients deviates from the full integration

At the long timescale, when NPP switches from being N limited to P limited, the approximated SOM N decreases faster than in the full numerical integration (Fig. 2E) and the approximated plant-available N (red line) does not increase as rapidly as in the full numerical

integration (blue dashed line; Fig. 2G). In the approximation SOM N dives to a quasi-equilibrium within the long timescale at a faster rate (approximately m_N, which is 1/33 per year) than the rest of the long timescale dynamics ((m_P(1-$\kappa_{\delta P}$) + φ_P) ≈ 1/1000 per year and ψ ≈ 1/4000 per year). Plant-available N on this timescale depends on the balance of N mineralization and plant uptake, and because SOM N (and thus N mineralization) decreases faster than plant biomass, the approximation is off from the full simulation for some time after the switch to P limitation.

At the intermediate timescale, which is N limited for this simulation, the approximated SOM P does not change, but it does in the full numerical integration (Fig. 2F). This leads to more plant-available P in the approximation (Fig. 2H) because the P mineralization flux is larger than in the full integration, when SOM P declines in the intermediate timescale.

Short timescale results

Litter N and P quasi equilibria and transient dynamics are independent of whether N or P is limiting, whereas plant-available N and P results depend on which nutrient limits NPP. Setting equation 2 to zero,

$$\hat{L}_i = \frac{(\mu + \theta_\mu F)B}{\omega_i(\delta_i + h_i)} \quad \text{(S1)}$$

where the hat above a variable indicates a quasi equilibrium value. Biologically, the quasi equilibrium for each nutrient is the N or P flux entering the litter pools via plant turnover multiplied by the mean litter N or P residence time (see below).

Integrating equation 2 yields the transient dynamics of litter N and P:

$$L_i(t) = \hat{L}_i + \left(L_i(0) - \hat{L}_i\right)e^{-(\delta_i + h_i)t} \quad \text{(S2)}$$

where $L_i(t)$ is the value of variable L_i at time t following a perturbation and $L_i(0)$ is its state immediately after the perturbation. Because the exponential terms are always negative (i.e., the δs and hs are all positive), equation S2 begins at $L_i(0)$ (which may be above or below their quasi equilibria) and approaches the quasi equilibrium value given in equation S1 in a saturating manner (Fig. S1A). The rate at which litter N or P approaches its quasi equilibrium is given by the exponential term in equation S2, $\delta_i + h_i$. Except in systems where ground fires or other litter loss mechanisms are very common, we expect litter decomposition to be much faster than litter loss from the ecosystem ($\delta_i >> h_i$), so the litter timescale for each nutrient i is approximately $1/\delta_i$, the inverse of the litter decomposition rate for that nutrient.

Available N and P dynamics depend on whether N or P limits NPP, so we present results for both, beginning with N limitation. From equations 4-6, the quasi equilibria for plant-available N and P when N limits NPP (with N limitation denoted by the subscript ",N" following the variable name) are

$$\hat{A}_{N,N} = \frac{I_N + m_N D_N + \dfrac{\theta_g}{\omega_N} BF + \varepsilon_N \delta_N \hat{L}_N}{k_N + B\nu_N}$$

$$\hat{A}_{P,N} = \frac{1}{k_P}\left(I_P + m_P D_P - \frac{(\omega_N - \theta_g)}{\omega_P} BF + \varepsilon_P \delta_P \hat{L}_P - \frac{\omega_N}{\omega_P} B\nu_N \hat{A}_{N,N} \right)$$

where, as above, $\hat{A}_{P,N}$ indicates the quasi-equilibrium for A_P if plants were N limited, and B, D_i, and I_P are treated as constants rather than variables.

Integrating equations 4 and 5 using the N limited part of equation 6 yields the following expression for the transient dynamics of A_N and A_P:

$$A_{N,N}(t) = \hat{A}_{N,N} + \left(A_N(0) - \hat{A}_{N,N} - C_{Ns1}\right) e^{-(k_N + B\nu_N)t} + C_{Ns1} e^{-(\delta_N + h_N)t}$$

$$A_{P,N}(t) = \hat{A}_{P,N} + \left(A_P(0) - \hat{A}_{P,N} - C_{Ns2} + C_{Ns3} - C_{Ns4}\right)e^{-k_P t}$$
$$+ C_{Ns2}e^{-(\delta_P + h_P)t} - C_{Ns3}e^{-(k_N + B\nu_N)t} + C_{Ns4}e^{-(\delta_N + h_N)t}$$

$$C_{Ns1} = \frac{\varepsilon_N \delta_N \left(L_N(0) - \hat{L}_N\right)}{k_N + B\nu_N - \delta_N - h_N}$$

$$C_{Ns2} = \frac{\varepsilon_P \delta_P \left(L_P(0) - \hat{L}_P\right)}{k_P - \delta_P - h_P}$$

$$C_{Ns3} = \frac{\frac{\omega_N}{\omega_P} B\nu_N \left(A_N(0) - \hat{A}_{N,N} - C_{Ns1}\right)}{k_P - k_N - B\nu_N}$$

$$C_{Ns4} = \frac{C_{Ns1}}{k_P - \delta_N - h_N}$$

Like the transient dynamics for litter N and P, available N and P begin at $A_N(0)$ and $A_P(0)$ and approach $\hat{A}_{N,N}$ and $\hat{A}_{P,N}$ because all the exponential terms are negative. However, unlike litter N and P there are not single controlling rates for available N and P. Available N has two controlling rates, $B\nu_N + k_N$ and $\delta_N + h_N$. Plant uptake is likely to be much faster than available N loss ($B\nu_N >> k_N$), so the controlling rates are approximately $B\nu_N$ and δ_N. If these two are approximately equal, available N goes from $A_N(0)$ to $\hat{A}_{N,N}$ with a single saturation rate, $B\nu_N + \delta_N$, although it can have one overshoot before saturating (see below). However, it is more likely that the plant uptake rate far exceeds the litter decomposition rate ($B\nu_N >> \delta_N$), in which case A_N has two controlling rates within our short timescale. It begins at $A_N(0)$ and approaches $\hat{A}_{N,N} + C_{Ns1}$ (where C_{Ns1} is the first constant for the N limited solutions to the short timescale) at the plant uptake rate ($B\nu_N$), then after quasi equilibrating at $\hat{A}_{N,N} + C_{Ns1}$ it proceeds to $\hat{A}_{N,N}$ at the litter N decomposition rate (δ_N). The sign of C_{Ns1} depends on whether litter N begins above or below its quasi equilibrium. Assuming the plant N uptake rate exceeds the litter decomposition rate, C_{Ns1} is positive if litter N begins above its quasi equilibrium and negative otherwise. For example, if

a perturbation removes litter but increases available N (e.g., a ground fire that combusts some litter N but also mineralizes some), C_{Ns1} is negative, so A_N drops from its initial large value $A_N(0)$ (which has been fertilized from the instant mineralization) to a low value, $\hat{A}_{N,N} + C_{Ns1}$ (a quasi equilibrium in which plants have taken up the excess mineralized N but the litter N pool has not yet recovered), then returns to $\hat{A}_{N,N}$ when litter returns to its quasi equilibrium (lower line in Fig. S1B, upper line in Fig. S1A). If instead the perturbation increases both available N and litter N (e.g., a combined litter addition and fertilization manipulation), there will still be two apparent saturations, but no oscillation (upper line in Fig. S1B, lower line in Fig. S1A).

Under the N limited scenario, available P has up to four controlling rates: the two it shares with available N as well as the litter P decomposition rate ($\delta_P + h_P \approx \delta_P$) and the available P loss rate (k_P). It follows the same course of multiple saturations as A_N, and thus can overshoot or approach the quasi equilibrium monotonically depending on whether the perturbation increases A_N, L_N, and L_P relative to their quasi equilibria (these determine the signs of C_{Ns1}, C_{Ns2}, C_{Ns3}, and C_{Ns4}). For example, a ground fire will mineralize but not combust litter P, decreasing L_N and L_P but increasing A_N and especially A_P relative to their quasi equilibria. The litter P decomposition rate is likely to be similar to the litter N decomposition rate, but the available P loss rate (k_P) might be larger or smaller. Many dynamics are possible here depending on various parameters, but as noted in the main text, the timescale approximations for the non-limiting nutrient (unlike the limiting nutrient) are not extremely accurate representations of the full system.

If P limits NPP at the short timescale, there are different expressions for the quasi equilibria and transient dynamics of A_N and A_P:

$$\hat{A}_{N,P} = \frac{1}{k_N}\left(I_N + m_N D_N + \left(1 + \frac{\theta_g}{\omega_N}\right) BF + \varepsilon_N \delta_N \hat{L}_N - \frac{\omega_P}{\omega_N} B \nu_P \hat{A}_{P,P} \right)$$

$$\hat{A}_{P,P} = \frac{I_P + m_P D_P + \dfrac{\theta_g}{\omega_P} BF + \varepsilon_P \delta_P \hat{L}_P}{k_P + B\nu_P}$$

$$A_{P,P}(t) = \hat{A}_{P,P} + \left(A_P(0) - \hat{A}_{P,P} - C_{Ps4}\right)e^{-(k_P + B\nu_P)t} + C_{Ps4}e^{-(\delta_P + h_P)t}$$

$$A_{N,P}(t) = \hat{A}_{N,P} + \left(A_N(0) - \hat{A}_{N,P} - C_{Ps1} + C_{Ps2} + C_{Ps3}\right)e^{-k_N t}$$
$$+ C_{Ps1}e^{-(\delta_N + h_N)t} - C_{Ps2}e^{-(k_P + B\nu_P)t} - C_{Ps3}e^{-(\delta_P + h_P)t}$$

$$C_{Ps1} = \frac{\varepsilon_N \delta_N \left(L_N(0) - \hat{L}_N\right)}{k_N - \delta_N - h_N}$$

$$C_{Ps2} = \frac{\dfrac{\omega_P}{\omega_N} B\nu_P \left(A_P(0) - \hat{A}_{P,P} - C_{Ps4}\right)}{k_N - k_P - B\nu_P}$$

$$C_{Ps3} = \frac{\dfrac{\omega_P}{\omega_N} B\nu_P C_{Ps4}}{k_N - \delta_P - h_P}$$

$$C_{Ps4} = \frac{\varepsilon_P \delta_P \left(L_P(0) - \hat{L}_P\right)}{k_P + B\nu_P - \delta_P - h_P}$$

Mathematically, the results when P limits NPP are similar to those for N limitation. Available P (the limiting nutrient) has two potential controlling rates, the plant P uptake rate ($B\nu_P + k_P \approx B\nu_P$) and the litter P decomposition rate ($\delta_P + h_P \approx \delta_P$), and available N (the non-limiting nutrient) has those two as well as potentially two more, the litter N decomposition rate ($\delta_N + h_N \approx \delta_N$) and the available N loss rate (k_N).

Limitation can switch from N to P or P to N on the short timescale. Immediately following a perturbation, limitation depends on which part of the minimum function in equation 6 is less when evaluated at $A_N(0)$, $A_P(0)$. For example, immediately following the application of N fertilizer NPP might be P limited, but if the short timescale quasi equilibrium is N limited then limitation would transition from P to N during the short timescale. In this case the transient

dynamics of available N and P exhibit kinks at the limitation switch on the short timescale. Limitation can switch during any of the saturations, but expressions describing this transition time are cumbersome and our focus is elsewhere so we omit them here.

In our simulation for Fig. 2, N is limiting at the end of the short timescale and plant available N begins farther from its quasi equilibrium than litter N or P do. Therefore, the time at which the short timescale transitions to the intermediate timescale, $t_{s\to m}$, is given by the time at which plant available N reaches 1% of its quasi equilibrium value. Assuming that $Bv_N + k_N >> \delta_N + h_N$ (approximately, plant uptake is faster than litter decomposition), this is given by

$$t_{s\to m} = \frac{-1}{\delta_N + h_N} \ln\left(\frac{0.01\hat{A}_{N,N}}{\left|C_{Ns1}\right|} \right)$$

Intermediate timescale results

The transient litter dynamics at the intermediate timescale are given by

$$\hat{L}_{i,N}\left(B_N(t)\right) \approx \hat{\hat{L}}_{i,N} + \left(\hat{\hat{L}}_{i,N} \left(\frac{B_N(0)}{\hat{B}_N} - 1 \right) \right) e^{-\mu'_N t},$$

where the intermediate timescale quasi equilibria expressions for litter N and P (with double hats) are the same as in the short timescale (equation S1) except that plant biomass is evaluated at its quasi equilibrium. For both litter N and P the single timescale is the N-limited plant biomass timescale, so they would have the same shape as plant biomass in Fig. 3A. As with equation 11, if μ'_N is negative plant biomass and litter N and P increase exponentially, whereas if it is positive they increase in a saturating manner like litter N and P on the short timescale.

The quasi equilibria and transient dynamics for available N and P are

$$\hat{\hat{A}}_{N,N} = \frac{\mu - (\omega_N - \theta)F}{\omega_N v_N}$$

$$\hat{\hat{A}}_{P,N} = \frac{1}{k_P}\left(I_P + m_P D_P - \frac{\mu'_P}{\omega_P}\hat{B}_N \right)$$

$$\mu'_P = \frac{(\mu + \theta_\mu F)(\delta_P(1-\varepsilon_P) + h_P)}{\delta_P + h_P}$$

$$\hat{A}_{N,N}(B_N(t)) \approx \frac{\theta_g F + \frac{\varepsilon_N \delta_N}{\delta_N + h_N}(\mu + \theta_\mu F)}{\omega_N \nu_N} + \frac{I_N + m_N D_N}{\nu_N B_N(t)}$$

$$\hat{A}_{P,N}(B_N(t)) \approx \frac{1}{k_P}\left(I_{(P-N)} - \frac{\omega_N}{\omega_P} B_N(t) F + \frac{\varepsilon_{(P-N)}}{\omega_P}(\mu + \theta_\mu F) B_N(t) \right)$$

$$I_{(P-N)} = I_P + m_P D_P - \frac{\omega_N}{\omega_P}(I_N + m_N D_N)$$

$$\varepsilon_{(P-N)} = \frac{\varepsilon_P \delta_P}{\delta_P + h_P} - \frac{\varepsilon_N \delta_N}{\delta_N + h_N}$$

where $\theta = \theta_\mu + \theta_g$.

The plant biomass quasi equilibrium and transient dynamics when N limits NPP (also given in the main text) are

$$\hat{B}_N = \frac{\omega_N}{\mu'_N}\left(I_N + m_N D_N - k_N \hat{\hat{A}}_{N,N} \right)$$

$$\mu'_N = \frac{(\mu + \theta_\mu F)(\delta_N(1-\varepsilon_N) + h_N)}{\delta_N + h_N} - \omega_N F$$

$$B_N(t) \approx \hat{B}_N + (B_N(0) - \hat{B}_N) e^{-\mu'_N t}$$

When P limits NPP, the expressions are as above but with the subscripts N and P reversed, except for available N:

$$\hat{A}_{N,P}(B_P(t)) \approx \frac{1}{k_N}\left(I_{(N-P)} + B_P(t) F - \frac{\varepsilon_{(P-N)}}{\omega_N}(\mu + \theta_\mu F) B_P(t) \right)$$

$$I_{(N-P)} = I_N + m_N D_N - \frac{\omega_P}{\omega_N}\left(I_P + m_P D_P\right)$$

The transition time from N to P limitation, if it occurs, is given by

$$t_{N\to P,m} = \frac{-1}{\mu'_N}\ln\left(\frac{-\left(B_N(0)-\hat{B}_N\right)}{2\left(a+b\hat{B}_N + c\hat{B}_N^2\right)}\left(-\left(b+2c\hat{B}_N\right)\pm\sqrt{b^2-4ac}\right)\right)$$

$$a = \omega_N\left(I_N + m_N D_N\right)$$

$$b = \left(\omega_N + \theta_g\right)F + \left(\frac{\varepsilon_N \delta_N}{\delta_N + h_N}\right)\left(\mu + \theta_\mu F\right) - \frac{\omega_P \nu_P I_{(P-N)}}{k_P}$$

$$c = \frac{\nu_P}{k_P}\left(\omega_N F - \varepsilon_{(N-P)}\left(\mu + \theta_\mu F\right)\right)$$

$$\varepsilon_{(N-P)} = \frac{\varepsilon_N \delta_N}{\delta_N + h_N} - \frac{\varepsilon_P \delta_P}{\delta_P + h_P}$$

Alternatively, if limitation switches from P to N during the intermediate timescale, the transition time is given by

$$t_{P\to N,m} = \frac{-1}{\mu'_P}\ln\left(\frac{-\left(B_P(0)-\hat{B}_P\right)}{2\left(a+b\hat{B}_P + c\hat{B}_P^2\right)}\left(-\left(b+2c\hat{B}_P\right)\pm\sqrt{b^2-4ac}\right)\right)$$

$$a = -\omega_P\left(I_P + m_P D_P\right)$$

$$b = \left(\omega_N - \theta_g\right)F - \left(\frac{\varepsilon_P \delta_P}{\delta_P + h_P}\right)\left(\mu + \theta_\mu F\right) + \frac{\omega_N \nu_N I_{(N-P)}}{k_N}$$

$$c = \frac{\nu_N}{k_N}\left(\omega_N F - \varepsilon_{(N-P)}\left(\mu + \theta_\mu F\right)\right)$$

The intermediate timescale transitions to the long timescale at the time when plant biomass reaches 1% of its quasi equilibrium, which is given by

$$t_{m\to l} = \frac{-1}{\mu'_N}\ln\left(\frac{0.01}{\left|\frac{B(0)}{\hat{B}_N}-1\right|}\right)$$

Long timescale results

The N limited equilibrium expressions are

$$\overline{B}_N = \frac{I_N - k_N\overline{A}_{N,N}}{\left(\frac{\mu+\theta_\mu F}{\omega_N}\right)\left(\frac{\delta_N\phi_N(1-\varepsilon_N)+h_N(m_N+\phi_N)}{(\delta_N+h_N)(m_N+\phi_N)}-F\right)}$$

$$\overline{L}_{i,N} = \frac{\mu+\theta_\mu F}{\omega_i(\delta_i+h_i)}\overline{B}_N$$

$$\overline{D}_{N,N} = \frac{(I_N - k_N\overline{A}_{N,N})\kappa_{\delta_N}}{m_N(1-\kappa_{\delta_N})+\phi_N}$$

$$\overline{D}_{P,N} = \frac{\delta_P(1-\varepsilon_P)(\mu+\theta_\mu F)}{\omega_P(\delta_P+h_P)(m_P+\phi_P)}\overline{B}_N$$

$$\overline{A}_{N,N} = \frac{\mu-(\omega_N-\theta)F}{\omega_N\nu_N}$$

$$\overline{A}_{P,N} = \frac{1}{k_P}\left(\alpha-\frac{1}{\omega_P}(\mu+\theta_\mu F)\left(\frac{\delta_P\phi_P(1-\varepsilon_P)+h_P(m_P+\phi_P)}{(\delta_P+h_P)(m_P+\phi_P)}\right)\overline{B}_N\right)$$

If P limits NPP on the long timescale, the equilibrium expressions are the same as for N limitation except the subscript N's and P's are reversed, I_N is the constant P input flux α, and terms with $\omega_N F$ or F on its own (but not θF or $\theta_\mu F$) are 0.

The N limited transient dynamics are

$$D_{N,N}(t) = \overline{D}_{N,N} + \left(D_{N,N}(0)-\overline{D}_{N,N}\right)e^{-\left(m_N(1-\kappa_{\delta_N})+\phi_N\right)t}$$

$$D_{P,N}(t) = \overline{D}_{P,N} + C_{Nl1}e^{-\left(m_N\left(1-\kappa_{\delta_N}\right)+\phi_N\right)t} + \left(D_{P,N}(0) - \overline{D}_{P,N} - C_{Nl1}\right)e^{-(m_P+\phi_P)t}$$

$$\hat{B}_N\left(D_{N,N}(t)\right) = \overline{B}_N + \frac{\omega_N m_N}{\mu'_N}\left(D_{N,N}(0) - \overline{D}_{N,N}\right)e^{-\left(m_N\left(1-\kappa_{\delta_N}\right)+\phi_N\right)t}$$

$$\hat{\hat{L}}_{N,N}\left(D_{N,N}(t)\right) = \overline{L}_{N,N} + \frac{\left(\mu+\theta_\mu F\right)m_N}{\left(\delta_N + h_N\right)\mu'_N}\left(D_{N,N}(0) - \overline{D}_{N,N}\right)e^{-\left(m_N\left(1-\kappa_{\delta_N}\right)+\phi_N\right)t}$$

$$\hat{\hat{L}}_{P,N}\left(D_{N,N}(t)\right) = \overline{L}_{P,N} + \frac{\omega_N\left(\mu+\theta_\mu F\right)m_N}{\omega_P\left(\delta_P + h_P\right)\mu'_N}\left(D_{N,N}(0) - \overline{D}_{N,N}\right)e^{-\left(m_N\left(1-\kappa_{\delta_N}\right)+\phi_N\right)t}$$

$$\hat{\hat{A}}_{N,N}\left(D_{N,N}(t)\right) = \overline{A}_{N,N}$$

$$\hat{\hat{A}}_{P,N}\left(D_{N,N}(t)\right) = \frac{1}{k_P}\left(\alpha + \gamma e^{-\psi t} + m_P D_{P,N}(t) - \frac{\mu'_P}{\omega_P}\hat{B}_N\left(D_{N,N}(t)\right)\right)$$

$$C_{Nl1} = \frac{\omega_N \kappa_{\delta_{P/N}} m_N\left(D_{N,N}(0) - \overline{D}_{N,N}\right)}{\omega_P\left(m_P + \phi_P - \left(m_N\left(1-\kappa_{\delta_N}\right)+\phi_N\right)\right)}$$

$$\kappa_{\delta_N} = \frac{\dfrac{\delta_N\left(1-\varepsilon_N\right)}{\delta_N + h_N}}{\dfrac{\delta_N\left(1-\varepsilon_N\right)+h_N}{\delta_N + h_N} - \dfrac{\omega_N F}{\mu + \theta_\mu F}} \tag{S3}$$

$$\kappa_{\delta_{P/N}} = \frac{\dfrac{\delta_P\left(1-\varepsilon_P\right)}{\delta_P + h_P}}{\dfrac{\delta_N\left(1-\varepsilon_N\right)+h_N}{\delta_N + h_N} - \dfrac{\omega_N F}{\mu + \theta_\mu F}}$$

In addition to sharing a controlling rate with SOM N, SOM P has another that is likely to be much faster, $m_P + \varphi_P$, which is approximately equal to m_P, the net mineralization rate of P in SOM. Plant-available P shares these two timescales, and also has a third controlling rate, ψ, the rock weathering rate. The rock weathering rate is likely to be substantially slower than φ_N. However, as noted above and on Fig. 2, these other timescales for the non-limiting nutrient do not play a significant role in the full system.

From the transient dynamics it is clear that the long timescale is globally stable if all the exponents are negative, which is true when

$$m_N\left(1-\kappa_{\delta_N}\right)+\phi_N > 0 \,. \tag{S4}$$

This is equivalent to the condition for stability for models of this general type [28]: losses of plant-unavailable N exceed N fixation inputs ($\overline{B}_N F < h_N \overline{L}_N + \phi_N \overline{D}_{N,N}$), as proven here. Plugging equation S3 into condition S4 and rearranging yields

$$\left(m_N+\phi_N\right)\left(\frac{\delta_N\left(1-\varepsilon_N\right)+h_N}{\delta_N+h_N}-\frac{\omega_N F}{\mu+\theta_\mu F}\right)>\frac{m_N\delta_N\left(1-\varepsilon_N\right)}{\delta_N+h_N}$$

Multiplying through by $\delta_N + h_N$, collecting terms, and canceling yield

$$\delta_N\phi_N\left(1-\varepsilon_N\right)+h_N\left(m_N+\phi_N\right)>\omega_N F\,\frac{\left(\delta_N+h_N\right)\left(m_N+\phi_N\right)}{\mu+\theta_\mu F}$$

Dividing both sides by the right hand side and multiplying by $\overline{B}_N F$ gives

$$\overline{B}_N\left(\mu+\theta_\mu F\right)\frac{\delta_N\phi_N\left(1-\varepsilon_N\right)+h_N\left(m_N+\phi_N\right)}{\omega_N\left(\delta_N+h_N\right)\left(m_N+\phi_N\right)}=h_N\overline{L}_{N,N}+\phi_N\overline{D}_{N,N}>\overline{B}_N F$$

When P limits NPP on the long timescale, the transient dynamics are

$$D_{N,P}(t)=\overline{D}_{N,P}+C_{Pl2}e^{-\psi t}+C_{Pl3}e^{-\left(m_P\left(1-\kappa_{\delta P}\right)+\phi_P\right)t}+\left(D_{N,P}(0)-\overline{D}_{N,P}-C_{Pl2}-C_{Pl3}\right)e^{-\left(m_N+\phi_N\right)t}$$

$$D_{P,P}(t)=\overline{D}_{P,P}+C_{Pl1}e^{-\psi t}+\left(D_{P,P}(0)-\overline{D}_{P,P}-C_{Pl1}\right)e^{-\left(m_P\left(1-\kappa_{\delta P}\right)+\phi_P\right)t}$$

$$\hat{B}_P\left(D_{P,P}(t)\right)=\overline{B}_P+\frac{\omega_P}{\mu'_P}Z$$

$$\hat{\hat{L}}_{N,P}\left(D_{P,P}(t)\right)=\overline{L}_{N,P}+\frac{\omega_P\left(\delta_P+h_P\right)}{\omega_N\left(\delta_P\left(1-\varepsilon_P\right)+h_P\right)\left(\delta_N+h_N\right)}Z$$

$$\hat{\hat{L}}_{P,P}\left(D_{P,P}(t)\right)=\overline{L}_{P,P}+\frac{1}{\delta_P\left(1-\varepsilon_P\right)+h_P}Z$$

$$\hat{\hat{A}}_{N,P}\left(D_{P,P}(t)\right)=\frac{1}{k_N}\left(I_N+m_N D_{N,P}(t)-\frac{\mu_N'}{\omega_N}\hat{B}_P\left(D_{P,P}(t)\right)\right)$$

$$\hat{\hat{A}}_{P,P}\left(D_{P,P}(t)\right)=\overline{A}_{P,P}$$

$$Z=\left(\gamma+m_P C_{Pl1}\right)e^{-\psi t}+m_P\left(D_{P,P}(0)-\overline{D}_{P,P}-C_{Pl1}\right)e^{-\left(m_P\left(1-\kappa_{\delta_P}\right)+\phi_P\right)t}$$

$$\kappa_{\delta_P}=\frac{\delta_P\left(1-\varepsilon_P\right)}{\delta_P\left(1-\varepsilon_P\right)+h_P}$$

$$C_{Pl1}=\frac{\kappa_{\delta_P}\gamma}{m_P\left(1-\kappa_{\delta_P}\right)+\phi_P-\psi}$$

$$C_{Pl2}=\frac{C_{Pl1}\omega_P\kappa_{\delta_{N/P}}m_N}{\omega_N\left(m_N+\phi_N-\psi\right)}$$

$$C_{Pl3}=\frac{\omega_P\kappa_{\delta_{N/P}}m_N\left(D_{P,P}(0)-\overline{D}_{P,P}-C_{Pl1}\right)}{\omega_N\left(m_N+\phi_N-\left(m_P\left(1-\kappa_{\delta_P}\right)+\phi_P\right)\right)}$$

In addition to sharing the two controlling rates with SOM P, SOM N and plant-available N have a third, faster rate, $m_N + \varphi_N \approx m_N$, the net N mineralization rate from SOM. As noted above, this faster rate is not noticeable in the full numerical integration. Plant-available P is constant on this long timescale.

The transition from N to P limitation at the long timescale occurs at the time $t_{N\rightarrow P,l}$ given by

$$0=a_1+\gamma e^{-\psi t_{N\rightarrow P,l}}+b_1 e^{-\left(m_P+\phi_P\right)t_{N\rightarrow P,l}}+c_1 e^{-\left(m_N\left(1-\kappa_{\delta_N}\right)+\phi_N\right)t_{N\rightarrow P,l}}$$

$$a_1=\alpha-k_P\overline{A}_{P,P}+m_P\overline{D}_{P,N}-\frac{\mu_P'}{\omega_P}\overline{B}_N$$

$$b_1=m_P\left(D_{P,N}(0)-\overline{D}_{P,N}-C_{Nl1}\right)$$

$$c_1=m_P C_{Nl1}-\frac{\mu_P'\omega_N}{\mu_N'\omega_P}m_N\left(D_{N,N}(0)-\overline{D}_{N,N}\right)$$

This equation cannot be solved explicitly because there are three distinct timescales involved. However, if the rates of DON loss (ϕ_N) and rock weathering (ψ) are sufficiently different, the exact time can be solved, as shown for the rock weathering transition time in the main text.
